# Conservation of *AtTZF1, AtTZF2*, and *AtTZF3* homolog gene regulation by salt stress in evolutionarily distant plant species

**DOI:** 10.3389/fpls.2015.00394

**Published:** 2015-06-16

**Authors:** Fabio D’Orso, Anna M. De Leonardis, Sergio Salvi, Agata Gadaleta, Ida Ruberti, Luigi Cattivelli, Giorgio Morelli, Anna M. Mastrangelo

**Affiliations:** ^1^Food and Nutrition Research Centre, Council for Agricultural Research and EconomicsRome, Italy; ^2^Cereal Research Centre, Council for Agricultural Research and EconomicsFoggia, Italy; ^3^Department of the Sciences of Agriculture, Food and Environment, University of FoggiaFoggia, Italy; ^4^Department of Soil, Plant and Food Sciences, “Aldo Moro” University of BariBari, Italy; ^5^Institute of Molecular Biology and Pathology, National Research CouncilRome, Italy; ^6^Genomics Research Centre, Council for Agricultural Research and EconomicsFiorenzuola d’Arda, Italy

**Keywords:** RR-TZF, CCCH zinc finger proteins, abiotic stress, germination, phylogenetic analysis, durum wheat, *Arabidopsis*, *Physcomitrella patens*

## Abstract

Arginine-rich tandem zinc-finger proteins (RR-TZF) participate in a wide range of plant developmental processes and adaptive responses to abiotic stress, such as cold, salt, and drought. This study investigates the conservation of the genes *AtTZF1-5* at the level of their sequences and expression across plant species. The genomic sequences of the two *RR-TZF* genes *TdTZF1-A* and *TdTZF1-B* were isolated in durum wheat and assigned to chromosomes 3A and 3B, respectively. Sequence comparisons revealed that they encode proteins that are highly homologous to AtTZF1, AtTZF2, and AtTZF3. The expression profiles of these RR-TZF durum wheat and *Arabidopsis* proteins support a common function in the regulation of seed germination and responses to abiotic stress. In particular, analysis of plants with attenuated and overexpressed AtTZF3 indicate that AtTZF3 is a negative regulator of seed germination under conditions of salt stress. Finally, comparative sequence analyses establish that the *RR-TZF* genes are encoded by lower plants, including the bryophyte *Physcomitrella patens* and the alga *Chlamydomonas reinhardtii*. The regulation of the *Physcomitrella AtTZF1-2-3-like* genes by salt stress strongly suggests that a subgroup of the RR-TZF proteins has a function that has been conserved throughout evolution.

## Introduction

Zinc-finger proteins constitute one of the largest and most diverse families of plant regulatory proteins, and increasing evidenc indicates that they act as key factors in several biological processes, such as morphogenesis and responses to environmental stress (Hall, 2005; Lin et al., 2011). These proteins are characterized by zinc-finger motifs that consist of cysteine and/or histidine residues that coordinate a zinc ion to form local peptide structures that are generally associated with specific molecular functions (Guo et al., 2009). The CCCH zinc-finger motif, in particular, identifies a specific zinc-finger family, members of which have been found in many species from yeast to human (Blackshear et al., 2005; Wang et al., 2008; Kramer et al., 2010). The gene family for these CCCH zinc-finger proteins has also been studied in plants, and various members have been identified by genome-wide analysis and have been implicated in a broad range of developmental and adaptive processes (Wang et al., 2008; Chai et al., 2012; Peng et al., 2012; Zhang et al., 2013; Liu et al., 2014; Xu, 2014).

A particular group of the plant CCCH protein family is characterized by an arginine-rich region that contains a CHCH motif (known as the RR region) that is linked to a tandem zinc-finger (TZF) domain, which is unique to plants (Wang et al., 2008; Pomeranz et al., 2010b). Members of the RR-TZF subfamily have been identified in several higher plants, including *Arabidopsis* (AtTZF1-11) (Wang et al., 2008; Chai et al., 2012; Peng et al., 2012; Zhang et al., 2013; Liu et al., 2014; Xu, 2014). The functions of some of the RR-TZF *Arabidopsis* proteins have been defined in relation to responses to a number of stress stimuli and developmental processes. PEI1 (AtTZF6) is involved in embryogenesis (Li and Thomas, 1998), and AtTZF10 and AtTZF11 (which are also known as AtSZF2 and AtSZF1, respectively) have been described as positive regulators of salt tolerance (Sun et al., 2007). The overexpression of AtTZF2 and AtTZF3, which are two RR-TZF genes that are regulated by abscisic acid (ABA), conferred ABA hypersensitivity, reduced transpiration, enhanced drought tolerance, and delayed jasmonic-acid-induced senescence, whereas their attenuation showed reduced tolerance to salt stress (Lee et al., 2012). Overexpression of another member of the RR-TZF subfamily, AtTZF1, resulted in late flowering and enhanced tolerance to cold and drought stress, while concurrent down-regulation of the AtTZF1-3 genes by RNA interference (RNAi) caused early germination and phenotypes that were relatively stress-sensitive (Lin et al., 2011). These studies have suggested a connection between AtTZF1 and ABA- and gibberellic-acid-mediated responses (Lin et al., 2011). The rice OsTZF1 gene is positively regulated by polyethylene glycol and ABA, and its overexpression in transgenic rice seedlings conferred hypersensitivity to ABA (Zhang et al., 2012). OsTZF1 has been reported to be involved in seed germination, seedling growth, leaf senescence, and oxidative-stress tolerance (Kong et al., 2006; Jan et al., 2013). In transgenic *Arabidopsis* plants, overexpression of TaZnFP, a *T. aestivum* gene that is induced by cold, salt, drought, and ABA, enhanced the *Arabidopsis* drought and salt tolerance (Min et al., 2013). Taken together, these data indicate a role for some of the RR-TZF proteins in ABA and gibberellic-acid pathway(s). Indeed, it has been established that the *Arabidopsis* SOMNUS (AtTZF4/SOM) protein acts as a negative regulator of light-dependent seed germination, and *som*-mutants are characterized by reduced levels of ABA and elevated levels of gibberellic acid, which appears to be due to changes in the expression of the ABA and gibberellic-acid metabolic genes (Kim et al., 2008). AtTZF4/SOM is positively regulated by the phytochrome-interacting factor-3-like 5 (PIL5) and ABA insensitive 3 (ABI3) transcription factors, which together bind to the AtTZF4/SOM promoter (Kim et al., 2008; Park et al., 2011).

A putative *RR-TZF* gene was previously identified in durum wheat as a cold-responsive EST (EST002H8). Interestingly, the expression of this *RR-TZF* gene was also modulated by drought in a developmental- and genotype-dependent manner (Mastrangelo et al., 2005; De Leonardis et al., 2007). To gain further insight into this putative *RR-TZF* gene and its regulation, we identified and characterized two homeologous genes, named *TdTZF1-A* and *TdTZF1-B* that correspond to EST002H8. Based on the sequence similarity, gene structure, and expression analysis, we established that *AtTZF1, AtTZF2,* and *AtTZF3* (indicated as *AtTZF1-2-3*) are putative orthologs of the durum wheat *TdTZF1-A* and *TdTZF1-B* genes. In addition, we showed that AtTZF3 is a negative factor in the control of *Arabidopsis* seed germination in the presence of salt. Finally, an evolutionary computational analysis indicated the presence of highly conserved AtTZF1-2-3-like proteins in phylogenetically distant species, such as the bryophyte *Physcomitrella patens.* Similar to the *Arabidopsis AtTZF1-5* and durum wheat *TdTZF1-A* and *TdTZF1-B* genes, the *Physcomitrella TZF* genes were shown to be regulated by salt stress. Taken together, our data suggest that the function of the AtTZF1-2-3-like proteins in the regulation of seed germination has emerged from their roles in pre-existing NaCl-stress signaling pathways that control growth and development in lower plants.

## Materials and Methods

### Cloning of Full-Length *TdTZF1-A* and *TdTZF1-B* Genomic and cDNA Sequences

Genomic DNA was extracted from durum wheat leaves of cv. ‘Creso’ using the cetyl trimethyl ammonium bromide (CTAB)-based method (Hoisington et al., 1994). Total RNA was isolated with Trizol reagent (Invitrogen), according to the manufacturer instructions. The single-stranded cDNA was synthesized from total RNA using SuperScript II RNase H reverse transcriptase (Invitrogen) and an oligod(T)_18_ primer, following the manufacturer recommendations.

A partial DNA sequence that codes for the RR-TZF protein (*EST002H8*) was used as a query in a BLASTN search with the database of the wheat separate chromosome arms promoted by the International Wheat Genome Sequencing Consortium^1^ (IWGSC), to gain the corresponding full-length genomic sequence. Two very similar hits were identified on chromosomes 3A and 3B (IWGSC_chr_3AS_ab_k71_contings_longerthan_200_3415369, and IWGSC_chr_3B_ab_k71 contings_longerthan_200_10407484). However, the genomic clones identified contained only partial sequences that corresponded to the 3^′^ part of *EST002H8*. A BLASTN search carried out against the Triticeae full-length CDS database (TriFLDB) using the *EST002H8* sequence as the query allowed the identification of a full-length cDNA sequence (AK330326) that was 100% identical to the partial sequence on chromosome 3A (IWGSC_chr_3AS_ab_k71_contings_longerthan 200_3415369). Specific primers were designed on the AK330326 sequence and used to amplify this gene in the durum wheat cultivar ‘Creso’, which we named as *TdTZF1-A*.

The full-length *TdTZF1-A* sequence was used as a query in a BLASTN search with the database of the wheat 3B chromosome promoted by the IWGSC. A new clone that showed 95% identity with respect to the query sequence was obtained (IWGSC_chr_3B_ab k71_contings_longerthan_200_10722038). However, this clone corresponded to a partial sequence; in particular, it contained the 5^′^ portion of the putatively homeologous gene of *TdTZF1-A*. Therefore, it was used together with the *EST002H8* sequence corresponding to the 3^′^ region of the gene to design specific primers to isolate *TdTZF1* on durum wheat genome B (*TdTZF1-B*). The forward primer was designed on the clone IWGSC_chr_3B_ab_k71_contings_longerthan_200_10722038 within the 5^′^-UTR region of the gene, and the reverse primer was designed on the *EST002H8* durum sequence. The bread-wheat cv. ‘Chinese Spring’ and the durum wheat cv. ‘Creso’ DNA and genomic sequences described in this study have been submitted to GenBank, with the accession numbers: KP717034-39.

### Physical Mapping of the *TdTZF1-A* and *TdTZF1-B* Genes in Heat

Nulli-tetrasomic lines of ‘Chinese Spring’ wheat for chromosomes 3A, 3B, and 3D (N3AT3D, N3BT3D, and N3D3B, respectively; Endo and Gill, 1996) were used to assign the *TdTZF1-A* and *TdTZF1-B* genes to specific chromosomes. Ditelosomic and deletion lines of chromosomes 3A and 3B were also used to further restrict the position of the *TdTZF1-A* and *TdTZF1-B* genes to specific bins. The primer pairs and PCR conditions were the same as those described for the cloning of the full-length sequences. The amplification products were separated on agarose gels and sequenced.

### Identification of Arginine-Rich Tandem Zinc-Finger Proteins in Plant Species

To identify the RR-TZF proteins in the plant kingdom, the genomes of 54 plant species that ranged from green algae to angiosperms were investigated. The AtTZF3 protein sequence IDAYSCDHFRMYDFKVRRCARGRSHDWTECPYAH was used as the query to search the phytozome database using the BLASTP program^2^. As gymnosperm genomes were not available in the phytozome database, the *Picea abies, Picea glauca, Picea sitchensis*, and *Pinus taeda* RR-TZF proteins were obtained from the Plant Transcription Factor Database^3^. The wheat RR-TZF proteins were obtained from the TriFLDB^4^, The Institute for Genomic Research (TIGR) Plant Transcript Assemblies^5^, the IWGSC^6^, and the National Center for Biotechnology Information^7^ (NCBI). The most similar proteins in each plant species were selected, and these were subsequently filtered based on the simultaneous presence of a CHCH motif, which is a distinctive feature of this subfamily (Wang et al., 2008), and TZF CCCH domains. The RR-TZF proteins from each species have been named according to the TZF nomenclature previously used by (Pomeranz et al., 2010a).

### Identification of the AtTZF1-2-3-like and AtTZF4-5-like Proteins

The AtTZF1-5 proteins are characterized by the absence of ANK repeats and by specific spacings between the Cys residues in the CCCH domains, which are typically C-X_7-8_-C-X_5_-C-X_3_-H for the first CCCH domain, and C-X_5_-C-X_4_-C-X_3_-H for the second CCCH domain. To search for putative orthologs of these proteins, a structure-based and homology-based approach was used: first, sequences structurally different from the AtTZF1-5 proteins were excluded (i.e., with ANK domains or with altered spacings between the Cys residues in the CCCH domains); then, for each sequence the most similar sequences in *Arabidopsis* were identified, based on a minimum similarity of 23%; and finally, these AtTZF1-2-3-like and AtTZF4-5-like proteins were clustered into two separate groups. Sequence similarities were obtained from the distance matrix of multi-alignments of all of the RR-TZF proteins.

### Alignments and Phylogenetic Analysis

All of the multiple alignments of amino-acid sequences were performed using the Clustal W algorithm in the Geneious software (version 5.5.3; Biomatters, New Zealand). The phylogenetic tree was constructed with the MEGA 5.0 software using the neighbor-joining method, after alignment of the RR-TZF full-length proteins from selected species through the Clustal W algorithm. For statistical reliability, bootstrap analysis with 1,000 replicates was used to evaluate the significance of each node.

### Plant Materials and Stress Conditions

#### Durum Wheat

*Triticum durum* cv. ‘Creso’ was used in this study. For all of the analyses, dry seeds were sterilized with 70% ethanol for 1 min, then with 3% sodium hypochlorite solution for 20 min, and finally washed five times with sterile water.

For the salt-stress gene expression analyses in germinating seeds, 15 seeds per dish were incubated on two layers of filter paper that had been moistened with distilled water, at 4°C in the dark for 72 h, to synchronize the germination. The seeds were moved to 21°C in the presence of light, with or without 150 mM NaCl. The germinating seeds were harvested for the expression analysis after 6 h and 12 h.

For the salt-stress gene expression analyses in seedlings, the sterilized wheat seeds were germinated and grown under controlled conditions (16 h photoperiod, at 100 μmol m^-2^ s^-1^ photon flux density) on two layers of filter paper that were moistened with distilled water. Then, the 4-day-old plantlets were transferred during light exposure to paper soaked with water or NaCl (at 150, 250, or 400 mM). The seedlings were sampled at the time of the transfer and after 1, 3, and 6 h.

For the cold-stress gene expression analyses, the sterilized wheat seeds were germinated and cultured on a mixture of soil, sand, and peat (6:3:1) in a growth chamber at 20°C (16 h light, at 500 μmol m^-2^ s^-1^ photon flux density)/ 18°C (8 h darkness), and 60% relative humidity, up to the full expansion of the third leaf. Then, the plants to be cold-stressed were moved to a chamber at 4°C (continuous light, at 500 μmol m^-2^ s^-1^ photon flux density), while the control plants were left under the initial conditions. The leaves were sampled at the time of the transfer and after 1, 3, and 6 h.

#### Arabidopsis thaliana

The *Arabidopsis thaliana* ecotype ‘Columbia’ (Col-0) was used in all of the experiments. The seeds were sterilized with 5% sodium hypochlorite solution that contained 0.02% Triton X-100, and then they were rinsed with water.

For the salt-stress gene expression analyses in germinating seeds, the sterilized seeds were spread on two layers of filter paper soaked with water or 150 mM NaCl and exposed to continuous light for 6 and 12 h. The material was collected from dry seeds and water-treated and salt-treated seeds.

For the salt-stress gene expression analyses in seedlings, the Col-0 seeds were spread on nylon sheets (Sefar Nitex 03-100/44) on germination medium (1x Murashige and Skoog salts, vitamins and 1% sucrose) solidified with 0.8% agar, and the seedlings were grown for 7 days at 21°C with a long photoperiod (16 h light, at 100 μmol m^-2^ s^-1^ photon flux density). Then, the nylon sheets were transferred onto the same substrate or onto germination media containing NaCl (150, 250, or 400 mM). Whole seedlings were collected at the time of the transfer and after 1, 3, and 6 h.

For the cold-stress gene expression analyses in seedlings, 7-day-old seedlings were grown on solid germination medium (0.8% agar) at 21°C with a long photoperiod (16 h light, at 100 μmol m^-2^ s^-1^ photon flux density) and were subjected to 10°C for 1, 3, and 6 h. The control seedlings were left at 21°C.

#### Physcomitrella patens

*Physcomitrella patens* ecotype ‘Gransden 2004’ was used in the experiments described in this study. The plants were grown at 21°C under a 16-h-light (40 μmol m^-2^ s^-1^)/8-h-dark photoperiod. Protonemata were propagated on a cellophane overlay on rich medium (BCD medium supplemented with 5% [w/v] glucose and 0.5 g/l ammonium tartrate; Roberts et al., 2011) solidified with 0.7% agar. After propagation, the protonemata were blended in water with a homogenizer. The homogenate was spread on a new cellophane disk on solid minimum BCD medium. After 6 days, the cellophane overlay with the protonemal tissue was transferred either onto the same substrate or onto media containing NaCl (150, 250, or 400 mM). Samples were collected at the time of the transfer and after 1, 3, and 6 h.

### Germination Tests

*Arabidopsis* wild-type and mutant (attenuated and over-expressing) AtTZF3 lines were propagated under controlled conditions in a growth room with a long photoperiod (16 h light, at 100 μmol m^-2^ s^-1^ photon flux density) at 21°C. Following seed harvesting, the seeds were dried at 21°C for at least 1 month prior to the germination assays. After sterilization, seeds were germinated on filter paper soaked with either water, 150 mM NaCl or 1 μM ABA solutions, at 21°C under continuous light (at 100 μmol m^-2^ s^-1^ photon flux density). The germination rate was scored after 72 h of incubation, with the emergence of a visible root used as the morphological marker for germination. Two experiments were carried out with two independent seed stocks, and each of these was performed using triplicate samples (each containing 50-100 seeds).

### Expression Analysis of *RR-TZF* Genes in Durum Wheat, *Arabidopsis*, and *Physcomitrella patens*

Total RNA was extracted from *T. durum*, *Arabidopsis* seedlings, and *P. patens* protonemata using RNeasy Plant Mini kits (Qiagen) with RNase-Free DNase (Qiagen) treatment, and from *Arabidopsis* and *T. durum* seeds using Spectrum plant total RNA kits (Sigma-Aldrich) with On-Coloumn DNase Digestion (Sigma-Aldrich). The RNA concentration and quality were determined spectrophotometrically (NanoDrop ND-1000 spectrophotometer, NanoDrop Technologies Inc., Montchanin, Germany).

The removal of any genomic DNA contamination from the total RNA and the first-strand cDNA synthesis were performed using QuantiTect Reverse Transcription kits (Qiagen), according to the manufacturer instructions, except for step 6, for which the incubation time was extended to 1 h.

Quantitative PCR was performed on an ABI7900HT PCR machine (Applied Biosystems), according to the manufacturer instructions, using TaqMan Universal PCR Master Mix (Applied Biosystems) and Universal ProbeLibrary Probes (Roche). The amplification reactions were carried out using a final Universal ProbeLibrary probe concentration of 100 nM, and a final primer concentration of 200 nM. The 384-well plates were set up using the Tecan Freedom Evo 75^®^ platform (Tecan). Each gene-specific expression quantification assay was designed using the free online ProbeFinder Roche software (www.universalprobelibrary.com), which allows an automated search for available probes, with annexed primers specific for the targets to be amplified. In some cases, the primer sequences were manually designed or just optimized. The primers and probes for each of the assays are listed in Supplementary Table S1. The expression levels of the reference gene (as indicated in each experiment) were used as the internal control. The relative expression level was calculated using the 2(-ΔΔCT) method (Livak and Schmittgen, 2001). The CT (cycle threshold) values used for both the target and the internal control genes were the means of three technical replicates. The experiments were performed at least twice, and the relative transcript levels were calculated and normalized as described previously (Willems et al., 2008).

### Construction of Attenuated and Overexpressing *Arabidopsis* Lines for the *AtTZF3* Gene

For the overexpression of *AtTZF3*, the *AtTZF3* open-reading frame was PCR amplified from genomic DNA and cloned into the 2x35S expression vector pMDC32 (Curtis and Grossniklaus, 2003).

Due to a lack of *AtTZF3* knock-out T-DNA insertions in mutant collections, RNAi, and artificial microRNAs (amiRNAs) were used to knock-down the expression of the gene. The amiRNA were generated by site-directed mutagenesis of the endogenous microRNA miR319a of *A. thaliana*, following the protocol described by Schwab et al. (2006), using modified primers (Supplementary Table S1). The RNAi was performed using an ihpRNA (intron hairpin RNA) constructs (Wesley et al., 2001), using the primers indicated in Supplementary Table S1.

The transformation of *Arabidopsis* was conducted according to the floral dip method (Clough and Bent, 1998), and single insertion T_3_ homozygous generation was used to determine the expression level of the *AtTZF3* transcript by RT-PCR.

### Statistical Analysis

Statistical analysis was performed after angular transformation of the germination ratios (Little and Hills, 1978), and after log2 transformation of the relative expression ratios. Statistical significance was evaluated by means of one-way ANOVA, followed by Bonferroni or Dunnett *post hoc* tests (Prism 6, GraphPad Software, CA, USA), as indicated in the Figure legends. *P* < 0.05 or < 0.01 were considered as statistically significant.

## Results

### Identification and Characterization of the *TdTZF1-A* and *TdTZF1-B* Genes in Durum Wheat

A partial DNA sequence coding for a RR-TZF protein named EST002H8 was previously identified as an abiotic stress-responsive gene in durum wheat (Mastrangelo et al., 2005; De Leonardis et al., 2007). To better characterize the genomic regions encoding EST002H8, the full-length sequences of two genes were isolated in the durum wheat cv. ‘Creso,’ which we named *TdTZF1-A* and *TdTZF1-B*. The genomic and cDNA sequences are identical, which indicated that no introns are present in these genes. *TdTZF1-A* and *TdTZF1-B* are 1,630-bp and 1,633-bp long, and they contain open-reading frames of 1,155 and 1,164 bp, respectively. The coded proteins are 384 and 387 amino-acids long, with calculated molecular weights of 41.17 kDa and 41.45 kDa, and isoelectric points of 7.53 and 7.51. TdTZF1-A and TdTZF-1B sequences show 89.5 and 96.4% identity at the nucleotide and amino-acid levels, respectively. The region containing the two CCCH domains is highly conserved, but the Ser-315 (polar) coded for by *TdTZF1-A* is replaced by the apolar Gly-319 coded for by *TdTZF1-B*. Moreover, the *TdTZF1-B* gene is characterized by a nucleotide insertion coding for three amino acids (i.e., serine, cysteine, glycine) at position 254, with respect to *TdTZF1-A* (Supplementary Table S2). *TdTZF1-A* and *TdTZF1-B* are putative homeologous genes, as a set of nulli-tetrasomic (i.e., N3AT3D, N3BT3D, N3DT3B) and deletion lines of bread wheat cv. ‘Chinese Spring’ and gene-specific primers allowed us to map the *TdTZF1-A* and *TdTZF1-B* sequences to the bins 3AS2-0.23-0.46 and C-3BS1-0.33 on chromosomes 3A and 3B, respectively (Supplementary Materials; Supplementary Figure S1).

**FIGURE 1:**
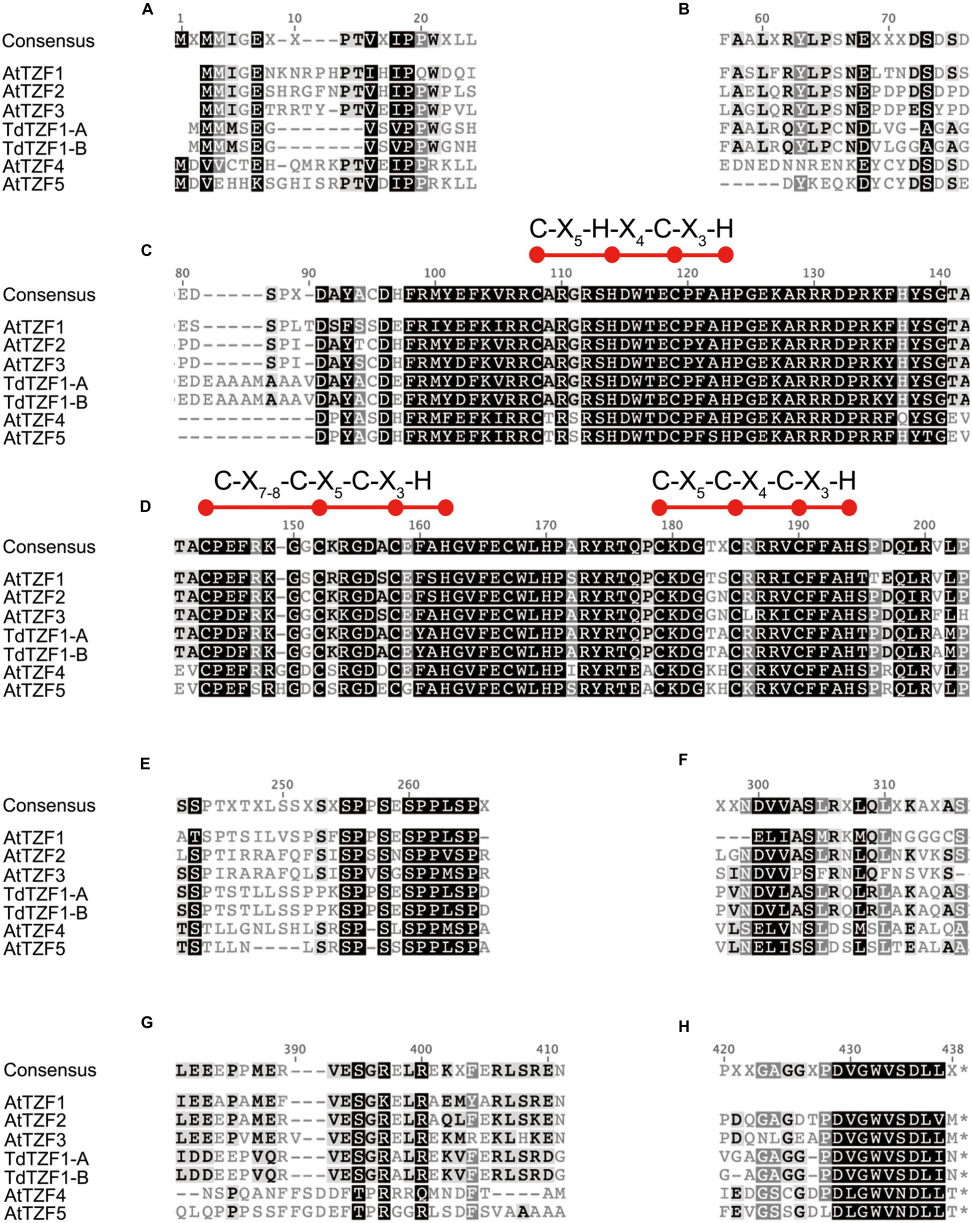
**Multi-alignment of the full-length amino-acid sequences of AtTZF1-5, TdTZF1-A, and TdTZF1-B.** Selected portions of the alignment of the AtTZF1-5, TdTZF1-A, and TdTZF1-B amino-acid sequences, constructed using the Clustal W algorithm with the Geneious software (version 5.5.3) **(A**-**H).** Black highlights amino acids that are identical (or analogous) in all of the sequences. Red balls and horizontal bars represent the CHCH motif or CCCH domains.

Sequence comparisons of TdTZF1-A and TdTZF1-B with the *Arabidopsis* AtTZF1-5 revealed that the wheat proteins are more similar to AtTZF1-2-3 than to AtTZF4-5 (**Figure 1**). Indeed, AtTZF1-2-3, TdTZF1-A, and TdTZF1-B have some amino-acid positions in common within or near the conserved RR-TZF region (e.g., Cys-95, Ala-109, Gly-111, Thr-141, Ala-142, Lys/Arg-153, Ala/Ser-157, Gln-177, Pro-178, Asp/Glu-197) that might discriminate the AtTZF1-2-3 group and TdTZF1-A and TdTZF1-B from the AtTZF4-5 group (**Figures 1C,D**). The gene structures (absence of introns) and the very high similarities of the TZF domains suggest that *AtTZF1-2-3*, *TdTZF1-A*, and *TdTZF1*-*B* are indeed orthologous genes. Moreover, there are some other motifs that are conserved in the grouping of AtTZF1-2-3, TdTZF1-A, and TdTZF1-B that are not in the AtTZF4-5 group (e.g., 60-LX[R/Q]YLP-65, **Figure 1B**; 381-[L/I]EEXPPMERVESGR-397, **Figure 1G**). Other motifs are common to both of these groups of proteins (e.g., 16-VXIPP-20, **Figure 1A**; 255-SPPSESPPLSP-265, **Figure 1E**; 299-NDVVASL-305, **Figure 1F**; 429-DVGWVSDLL-437, **Figure 1H**).

### *In Silico* Analysis of the *AtTZF1-5*, *TdTZF1-A*, and *TdTZF1-B* Genes during Development and Under Abiotic-Stress Conditions

As a first step to investigate whether there are any evolutionarily conserved features in the regulation of these genes, an *in silico* analysis of their expression profiles during plant development and in response to abiotic stress was performed (Supplementary Figure S2). The *AtTZF1-5* genes have well-defined counterparts in species in which the RR-TZF family has been described (Wang et al., 2008, 2014; Chai et al., 2012; Peng et al., 2012; Bogamuwa and Jang, 2014; Liu et al., 2014; Wang et al., 2014; Xu, 2014), and these were selected for this analysis. Furthermore, we identified other genes that belong to the *RR-TZF* family in wheat using an *in silico* search in which the *Arabidopsis* genes and the durum wheat *TdTZF1-A* and *TdTZF1-B* genes were used as queries against the TIGR, TriFLDB, NCBI, and IWGSC databases. As well as the bread-wheat AK330326 sequence, which is named here as *TaTZF1*, and a recently described bread-wheat gene, *TaZnFP* (*TaTZF2*) (Min et al., 2013), we retrieved six more CCCH sequences, as: AK335344 (*TaTZF3*), AK335750 (*TaTZF4*), and Tplb0012e12 (*TaTZF7*) in the TriFLDB database; EMS63094 (representing a *T. urartu* gene) in the NCBI; TC435797 (*TaTZF5*), as the only tentative consensus that corresponded to a full-length *TZF* gene in the wheat gene index database; and Ta377176 (*TaTZF6*) of 9456 bp, which was contained within IWGSC_chr2BS_ab_k71_contigs_longerthan_200_5175761. All of these sequences were blasted against the IWGCS database to predict their putative chromosome locations. This information was obtained for *TaTZF4* (chromosome 2AS), *TaTZF6* (chromosome 2BS), *TaTZF3* (chromosome 1DS), *TaZnFP* (chromosome 3B), and *TaTZF5* (chromosome 1AL). The *in silico* expression analysis was conducted using the following public databases: the *Arabidopsis* eFP Browser^8^ and PLEXdb^9^ for *Arabidopsis* and wheat, respectively. The wheat *RR-TZF* sequences were blasted against the database of PLEXdb to identify the probe set that corresponded to each gene. The same probe set was found for the *TaTZF1*, *TdTZF1-A*, and *TdTZF1-B* genes.

The *AtTZF2-3* genes showed the highest expression levels in seeds and senescent leaves, whereas a more specific expression in seeds was observed for *AtTZF1*-*4*-*5* (Supplementary Figure S2A). Similar behavior was seen for the wheat *TdTZF1-A* and *TdTZF1*-*B* genes (Supplementary Figure S2C). Then, we revealed a common feature of these genes: that they are regulated by abiotic stress. Clear overexpression was observed for *AtTZF1-3* in response to cold, salt, and osmotic stress, in both leaves and roots (Supplementary Figure S2B), whereas *TaTZF1*, *TdTZF1-A, TdTZF1-B*, and *TaTZF5* were all up-regulated by drought and cold stress (Supplementary Figure S2D,E).

To compare the expression profiles of these putative orthologous genes more closely, we analyzed the accumulation of the *AtTZF1-5*, *TdTZF1-A*, and *TdTZF1-B* transcripts in response to cold and salt stress, and during seed germination.

### Expression Profiles of the *TdTZF1-A* and *TdTZF1-B* Genes and *Arabidopsis* Homologs under Cold-Stress Conditions

**FIGURE 2:**
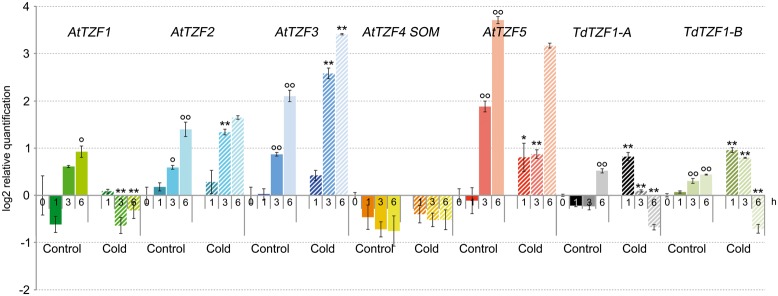
**Expression of the *AtTZF1-5*, *TdTZF1-A*, and *TdTZF1-B* genes under cold-stress conditions.** *Arabidopsis thaliana* and *T. durum* seedlings were grown at 21°C and then maintained at the same temperature or subjected to cold stress for different times. The relative expression levels are shown for *AtTZF1-5*, *TdTZF1-A*, and *TdTZF1*-*B* under control conditions and upon cold stress. Relative mRNA expression levels were referred to the untreated controls (0 h). Data are means of relative quantification (Log2) of three biological replicates normalized to *AtACT2* or *Td_Polyubiquitin* for the *A. thaliana* and *T. durum* target genes, respectively (±SE). Statistical significance was assessed by one-way ANOVA analysis followed by Bonferroni’s tests. °*P* < 0.05, ^∘∘^*P* < 0.01 1 h control, 3 h control, 6 h control vs 0 h control; ^∗^*P* < 0.05, ^∗∗^*P* < 0.01 1 h cold vs 1 h control, 3 h cold vs 3 h cold, 6 h cold vs 6 h control.

The expression profiles of *Arabidopsis AtTZF1-5* and the durum wheat genes *TdTZF1-A* and *TdTZF1-B* were investigated using RT-qPCR in 7-day-old seedlings exposed to low temperature for 1, 3, and 6 h (**Figure 2**). At 21°C, excluding *AtTZF4*/*SOM*, the *Arabidopsis* and durum wheat genes showed marked variations in transcript accumulation (up to 1.9-fold at 6 h) during the experimental time course, which suggested that their expression changed in response to light or the circadian cycle. For this reason, all of the experiments were initiated at the same time of day. The shift to lower temperature affected the expression profiles of all of the genes compared to controls, again except for *AtTZF4/SOM*. In particular, *AtTZF1* showed clear repression by low temperature (about 2.4-fold repression at 3 h); *AtTZF3* showed up-regulation in response to low temperatures at each time point, with a maximum of induction of 3.3-fold at 3 h; *AtTZF2* was up-regulated transiently (about 1.7-fold) 3 h from the beginning of the cold period. In contrast, *AtTZF5* showed the opposite behavior, as it was transiently down-regulated about twofold by the cold after 3 h of the low-temperature exposure.

Slight up-regulation during the cold treatment was observed for the durum wheat genes during the experimental time course. In particular, with the experimental set-up that was used here, both the *TdTZF1-A* and *TdTZF1-B* genes were transiently up-regulated about twofold by the cold treatment and then clearly down-regulated about 2.2-fold after 6 h of low-temperature exposure. However, neither of these two durum wheat genes showed clear overlapping expression profiles with any of the putative orthologous *Arabidopsis* genes.

### Expression Profiles of the *Arabidopsis* Homologs and the *TdTZF1-A* and *TdTZF1-B* Genes under Salt-Stress Conditions

**FIGURE 3:**
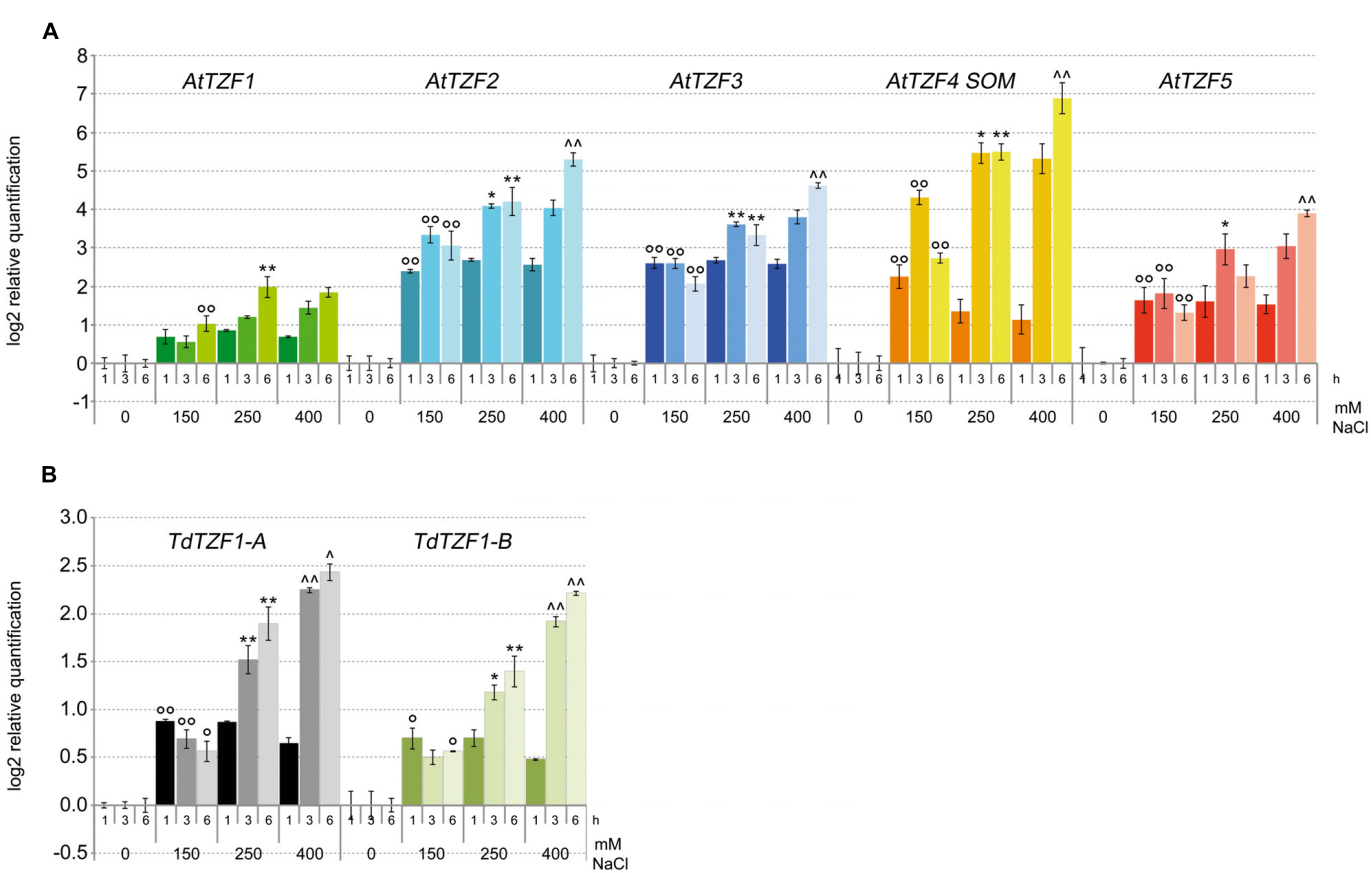
**Expression of the *AtTZF1-5*, *TdTZF1-A*, and *TdTZF1-B* genes under salt-stress conditions.** *Arabidopsis* **(A)** and *T. durum* **(B)** seedlings were grown in the absence of salt, and then treated with no salt or 150, 250, or 400 mM NaCl for different times. The relative expression levels are shown for *AtTZF1-5* **(A)**, *TdTZF1-A*, and *TdTZF1-B* **(B)** under control conditions and upon exposure to salt stress for different times. The relative mRNA expression levels of each salt-treated sample were referred to the corresponding time point of the control experiment (no NaCl). Data are means of relative quantification (Log2) of two (*T. durum*) or three (*A. thaliana*) biological replicates normalized to *AtACT2* or *Td_Polyubiquitin* for the *A. thaliana* and *T. durum* target genes, respectively (±SE). Statistical significance was assessed by one-way ANOVA analysis followed by Bonferroni’s tests. °*P* < 0.05, ^∘∘^*P* < 0.01 1 h 150 mM vs 1 h 0 mM, 3 h 150 mM vs 3 h 0 mM, 6 h 150 mM vs 6 h 0 mM; ^∗^*P* < 0.05, ^∗∗^*P* < 0.01 1 h 250 mM vs 1 h 150 mM, 3 h 250 mM vs 3 h 150 mM, 6 h 250 mM vs 6 h 150 mM; ^∧^*P* < 0.05, ^∧∧^*P* < 0.01 1 h 400 mM vs 1 h 250 mM, 3 h 400 mM vs 3 h 250 mM, 6 h 400 mM vs 6 h 250 mM.

Based on the data from the *in silico* expression analysis, we then determined the expression profiles of *AtTZF1-5*, *TdTZF1-A*, and *TdTZF1-B* in response to salt stress. The analysis was performed with the *Arabidopsis* and durum wheat seedlings exposed to different concentrations of NaCl (i.e., 0, 150, 250, 400 mM) for 1, 3, and 6 h (**Figure 3**). All of the analyzed genes showed rapid increases in their transcript levels in response to the salt stress, which were evident already at 1 h after the onset of the treatment. Clear dose-dependent regulation was seen in the responses to the increasing salt concentrations for all of these genes. However, *AtTZF2-4* showed a greater than twofold increases over the controls, which demonstrated stronger regulation with respect to *TdTZF1-A* and *TdTZF1-B*.

### Expression Profiles of the *Arabidopsis* Homologs and the *TdTZF1-A* and *TdTZF1-B* Genes in Dry Seeds and during Germination

**FIGURE 4:**
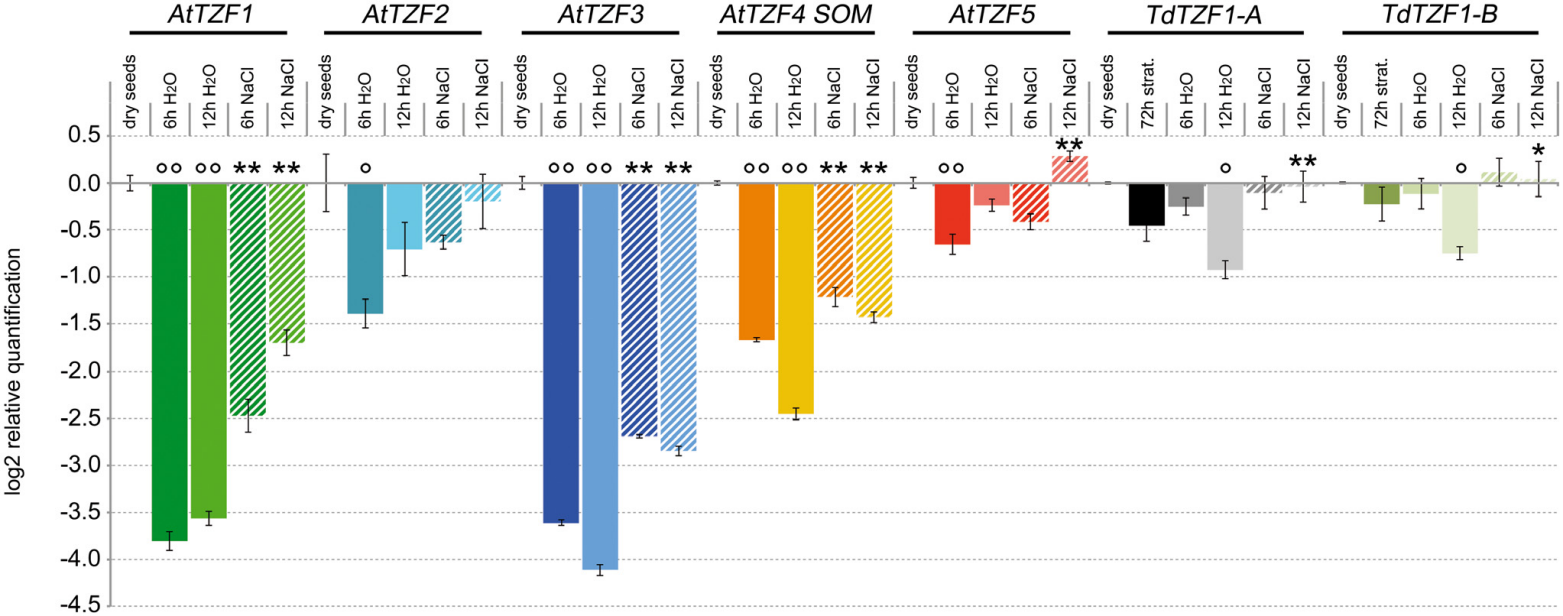
**Expression of the *AtTZF1-5*, *TdTZF1-A*, and *TdTZF1*-*B* genes during the germination process.** *Arabidopsis* and *T. durum* seeds were germinated in water or in the presence of 150 mM NaCl. The relative expression levels are shown for *AtTZF1-5*, *TdTZF1-A*, and *TdTZF1*-*B* in germinating seeds in water and in NaCl solution after 6 h and 12 h from the onset of germination. The relative expression levels are also shown for *TdTZF1-A* and *TdTZF1*-*B* in stratified seeds. Relative mRNA expression levels were referred to the dry seeds. Data are means of relative quantification (Log2) of three biological replicates normalized to *AtTIP41-like* and *Td_17S1* for the *A. thaliana* and *T. durum* target genes, respectively (±SE). Statistical significance was assessed by one-way ANOVA analysis followed by Bonferroni’s tests. °*P* < 0.05, ^∘∘^*P* < 0.01 72 h strat., 6 h H_2_O, 12 h H_2_O *vs* dry seeds; ^∗^*P* < 0.05, ^∗∗^*P* < 0.01 6 h NaCl vs 6 h H_2_O, 12 h NaCl vs 12 h H_2_O.

The analysis of gene expression conducted *in silico* (Supplementary Figure S2) suggested that the *RR-TZF* genes are actively transcribed in seeds. On the basis of these data, the expression profiles of the *AtTZF1-5*, *TdTZF1-A*, and *TdTZF1-B* genes were also investigated during the germination process (**Figure 4**). Interestingly, all of these genes showed very similar expression profiles. The steady-state levels of the transcripts in germinating seeds was always lower than in dry seeds, with the lowest levels reached at 6 h from the beginning of the germination process for *AtTZF2* (2.6-fold repression), *AtTZF1* (14-fold repression), and *AtTZF5* (1.5-fold repression), and at 12 h from the beginning of the germination process for *AtTZF3* (17.3-fold repression), *AtTZF4* (1.9-fold repression), *TdTZF1-A,* and *TdTZF1-B* (1.7- and 5.5-fold repression, respectively).

The effect of salt in the delaying of the germination process in both *Arabidopsis* and wheat is well known. Considering that in these seedlings the *RR-TZF* genes are regulated by salt (see **Figure 3**), we also analyzed their expression during the germination process in the presence of 150 mM NaCl. Interestingly, the decrease in the transcript levels of these *RR-TZF* genes during germination was lower in the presence of 150 mM NaCl than in water (**Figure 4**). In particular, *TdTZF1-A* and *TdTZF1-B* showed strong differences in their transcript accumulation after 12 h of salt treatment, with respect to the controls (about 1.8-fold), while all of the *Arabidopsis* genes showed differences between the NaCl-treated and untreated seeds even at 6 h after the beginning of the germination process (**Figure 4**). In analogy with the role of light, which promotes germination by repressing the negative regulation of *AtTZF4*/*SOM* on this process, these data suggested that salt increases the expression of *AtTZF1-5*, which results in a delay of seed germination.

### *AtTZF3* is a Negative Regulator of Seed Germination in the Presence of NaCl and Abscisic Acid

The results of the expression analysis shown in **Figure 4** suggested that the *Arabidopsis* AtTZF1-5 proteins and their homologs TdTZF1-A and TdTZF1-B have redundant actions as negative regulators of seed germination in the presence of salt. To test this hypothesis, we generated *Arabidopsis* lines that expressed either lower or higher levels of the *AtTZF3* gene.

**FIGURE 5:**
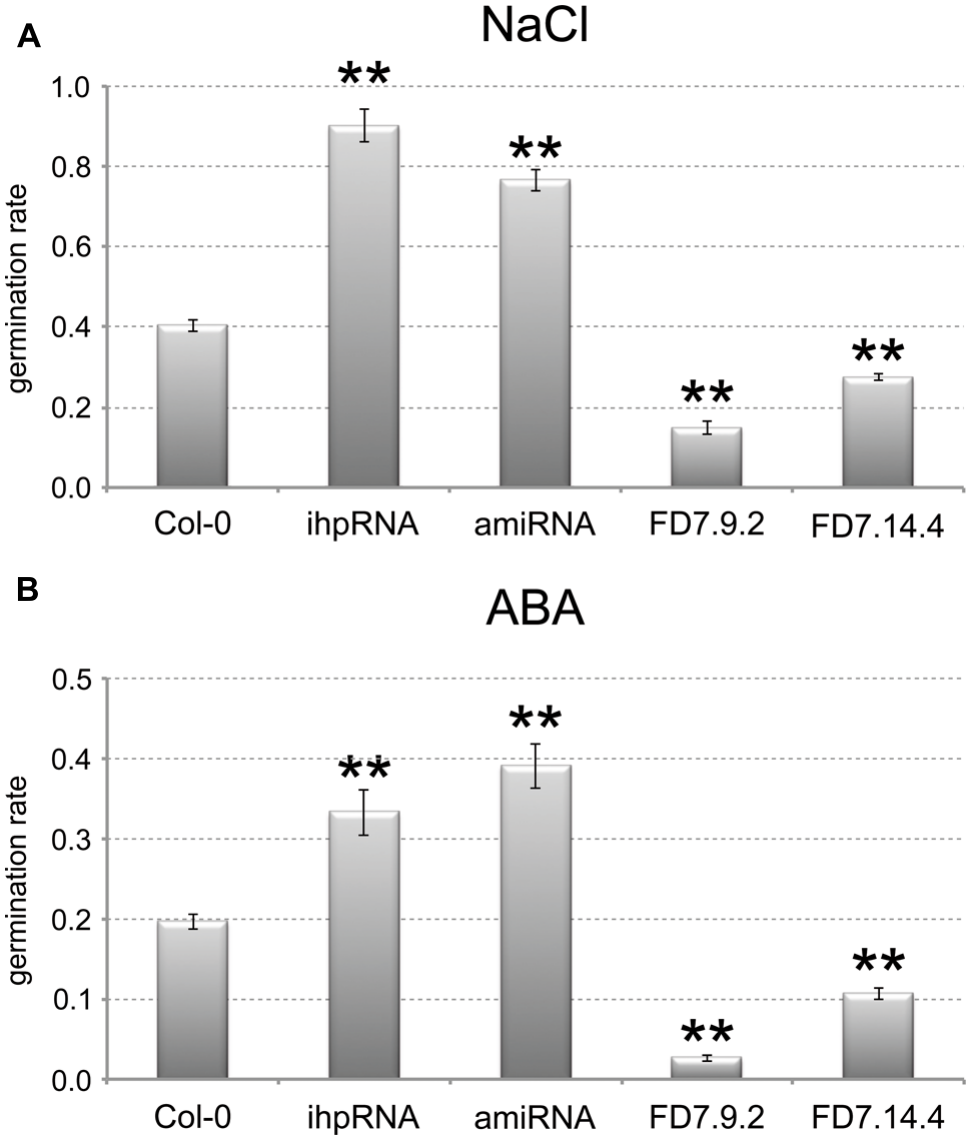
**AtTZF3 is a negative regulator of seed germination.** The germination rates are shown for Col-0, for two attenuated lines for AtTZF3 (ihpRNA, amiRNA), and for two independent lines overexpressing AtTZF3 (FD7.9.2, FD7.14.4) in the presence of 150 mM NaCl **(A)** or 1 μM ABA **(B)**. Data are means of three biological replicates (±SD), each one conducted with 50-100 seeds per genotype. Statistical significance was determined by one-way ANOVA analysis followed by Dunnett’s tests. ^∗^*P* < 0.05, ^∗∗^*P* < 0.01 ihpRNA, amiRNA, FD7.9.2 and FD7.14.4 vs Col-0.

Two *AtTZF3* knocked-down lines showed relevant reduction in the *AtTZF3* transcript (by up to 90%; data not shown): pK35.4 (ihpRNA), and pB23.3 (amiRNA). Two *AtTZF3* overexpressing lines showed high levels of *AtTZF3* mRNA (about 200-fold and 400-fold higher, respectively, compared with wild-type; data not shown): FD7.9.2 and FD7.14.4. These were assessed for their germination under normal conditions and upon treatment with NaCl and ABA. When these seeds were germinated with water, there were no differences in the germination rates between the Col-0 seeds and the transgenic lines with altered levels of *AtTZF3* expression (data not shown). Interestingly, completely different behavior was shown by these transgenic lines when NaCl was present in the growth medium. Indeed, the *AtTZF3* attenuated (i.e., pK35.4, pB23.3) and the *AtTZF3* overexpressing (i.e., FD7.9.2, FD7.14.4) lines showed opposite responses to salt, as more tolerant and more sensitive, respectively (**Figure 5A**).

Based on the consideration of the negative role of ABA in the germination process, we next tested the sensitivity of the transgenic lines to ABA. In analogy with NaCl, in terms of the germination rates, the attenuated lines were less sensitive and the overexpressing lines were more sensitive to 1 mM ABA treatment (**Figure 5B**). These germination trials in the presence of NaCl and ABA were replicated several times using different batches of seeds, and similar data were obtained (data not shown).

Taken together, these data suggested that *AtTZF3* is involved in NaCl-mediated and ABA-mediated regulation of seed germination.

### Identification of RR-TZF Proteins in Other Plant Species

Although many *RR-TZF* genes have been described, a complete survey and classification of all of the *RR-TZF* genes in plant species from disparate evolutionary groups is lacking. The completion of several high-quality plant-genome sequencing projects provided us with the unique opportunity to carry out a complete assessment and thorough comparative analysis of the plant RR-TZF proteins. As shown in **Figure 1**, the entire RR-TZF region is very well conserved between the *Arabidopsis* and wheat RR-TZF proteins. However, the conservation is particularly high both in the region upstream of the CCCH domains and in the CCCH domains themselves. Therefore, to establish the evolutionary conservation of this gene family across the plant kingdom, we used the AtTZF3 sequence IDAYSCDHFRMYDFKVRRCARGRSHDWTECPYAH, which includes the CHCH motif and some upstream amino acids, as the query in BLASTP searches to find the most similar sequences from several plant species. The sequences obtained were filtered using the presence of both the CHCH motif and the TZF CCCH domains. Through this, we defined 461 RR-TZF sequences, including several in lower plants and algae. The few proteins that showed high similarity with the query sequence but that lacked one or more of the requirements (i.e., the CHCH motif or the CCCH domain) were collected separately (Supplementary Table S3). These proteins are likely to have diverse or compromised functionality and/or to be encoded by pseudogenes, and they were found in *Coccomyxa subellipsoidea, P. patens, Capsella grandiflora, Amborella trichopoda, Glycine max, Zea mays, Malus domestica, Medicago truncatula*, and *Solanum lycopersicum*. The species analyzed and all of the sequences found are listed in Supplementary Tables S2 and S4, where the proteins are named according to the TZF nomenclature, with the indication of the structure and sequence of the CCCH domains and CHCH motifs, and the presence and type of ANK repeats.

For the first CCCH domain, in addition to the proteins with the conventional C-X_7-8_-C-X_5_-C-X_3_-H structure, several proteins that showed a more variable structure were found (i.e., altered spacing between the first and second Cys) (Supplementary Table S2). Several new sequences with a diverging CCCH domain were also identified in species in which the RR-TZF proteins have already been described, such as for *Z. mays*, *M. truncatula*, and *S. lycopersicum* (e.g., ZmTZF13, MtTZF6-14, SlyTZF9-12). In particular, ZmTZF13 contains 20 amino-acid residues between the first and the second Cys of the first CCCH domain. Although the amino-acid sequence C-X_20_-C-X_5_-C-X_3_-H would not be classified as a CCCH domain based on the definition by Wang et al. (2008), the high homology with the other RR-TZF proteins suggests that ZmTZF13 is indeed a member of the RR-TZF family. Therefore, the first CCCH domain might be better defined as the following more general structure: C-X_5-20_-C-X_5_-C-X_3_-H. The C-X_9_-C-X_5_-C-X_3_-H domains were found across proteins of several lineages, such as for monocotyledons (e.g., *T. aestivum*), dicotyledons (e.g., *A. lyrata, Capsella rubella, Linum usitatissimum*) and gymnosperms (e.g., *P. abies*, *P. glauca*) (Supplementary Table S2). The C-X_5_-C-X_5_-C-X_3_-H domains were found only in algal proteins (e.g., *Chlamydomonas reinhardtii*, *Volvox carteri*), whereas the C-X_10-20_-C-X_5_-C-X_3_-H domains were exclusively found in monocotyledon proteins (e.g., *Brachypodium distachyon, Oryza sativa, Panicum hallii, Panicum virgatum, Sorghum bicolor, T. aestivum*, *Z. mays*). The second CCCH domain, which was typically C-X_5_-C-X_4_-C-X_3_-H, is highly conserved in structure among all of the proteins, with the exception of two soybean (i.e., GmTZF17, GmTZF18) and two apple (i.e., MdTZF4, MdTZF16) proteins that are characterized by variant motifs (i.e., C-X_4_-C-X_4_-C-X_3_-H, C-X_7_-C-X_4_-C-X_3_-H, respectively).

Based on the sequence homology, we constructed a dendrogram that defined five distinct clusters of RR-TZF proteins (Supplementary Figure S3A). The largest group (i.e., RR-TZF I) corresponds to the proteins that are homologous to AtTZF7-11, and these are characterized by the presence of ANK repeats. The second group (i.e., RR-TZF II) contains the RR-TZF proteins that are highly homologous to AtTZF1-5; these proteins do not have ANK domains and they are relatively small (250 amino acids, on average). The third group (i.e., RR-TZF III) consists of proteins that are homologous to AtTZF6. Although these proteins are structurally similar to those of the AtTZF1-5 group, their amino-acid sequences are sufficiently different to form a separate group. Then the fourth group (i.e., RR-TZF IV) consists of proteins that are encoded by the gymnosperms and Solanum, and finally the fifth group (i.e., RR-TZF V) consists of proteins from monocotyledons (characterized by a larger spacing between the first and second Cys residues of the first CCCH domain) and algae. Interestingly, the multiple sequence alignments of the full-length RR-TZF proteins belonging to these five groups highlighted the presence of distinctive invariant residues in the RR-TZF region of all of the plant proteins (i.e., for monocotyledons, eudicotyledons, *Selaginella*, and *Physcomitrella*, and even among the unicellular green algae). Supplementary Figure S3B shows a schematic representation of the sequence conservation of the amino-acid residues, and the consensus sequence of the RR-TZF region. In addition, it is worth noting that the presence of several invariant amino-acid residues resulted in a signature that uniquely identifies the plant RR-TZF proteins (Supplementary Figure S3B).

### Identification of Genes Orthologous to *AtTZF1-3*, *TdTZF1-A*, and *TdTZF1-B*

To search for the *AtTZF1-3*, *TdTZF1-A*, and *TdTZF1-B* orthologs, we performed a comparative analysis according to a sequence-homology-based approach (see Materials and Methods). Here, we identified only two subgroups: the first subgroup (i.e., RR-TZF IIa) includes 117 AtTZF1-2-3-like sequences, and the second subgroup (i.e., RR-TZF IIb) consists of 49 AtTZF4-5-like proteins (Supplementary Table S5).

The AtTZF1-2-3-like proteins were found at all levels of the evolutionary scale, which included the green algae *C. subellipsoidea C-169* (CsTZF1), which thus suggested that their origin is very ancient. Moreover, even if their sequences show low similarity to the AtTZF1-2-3 proteins, some other RR-TZF proteins in green algae (e.g., CreTZF6, VcTZF6) might be orthologous to proteins of the RR-TZF IIa subgroup, as they share some specific amino-acid residues with them in the RR-TZF region (data not shown). The AtTZF4-5-like proteins were found only in angiosperm dicotyledon plants and in *A. trichopoda*, which is considered as the most primitive angiosperm, but not in monocotyledons. This indicates that their origin is more recent and that they are lineage-specific proteins.

**FIGURE 6:**
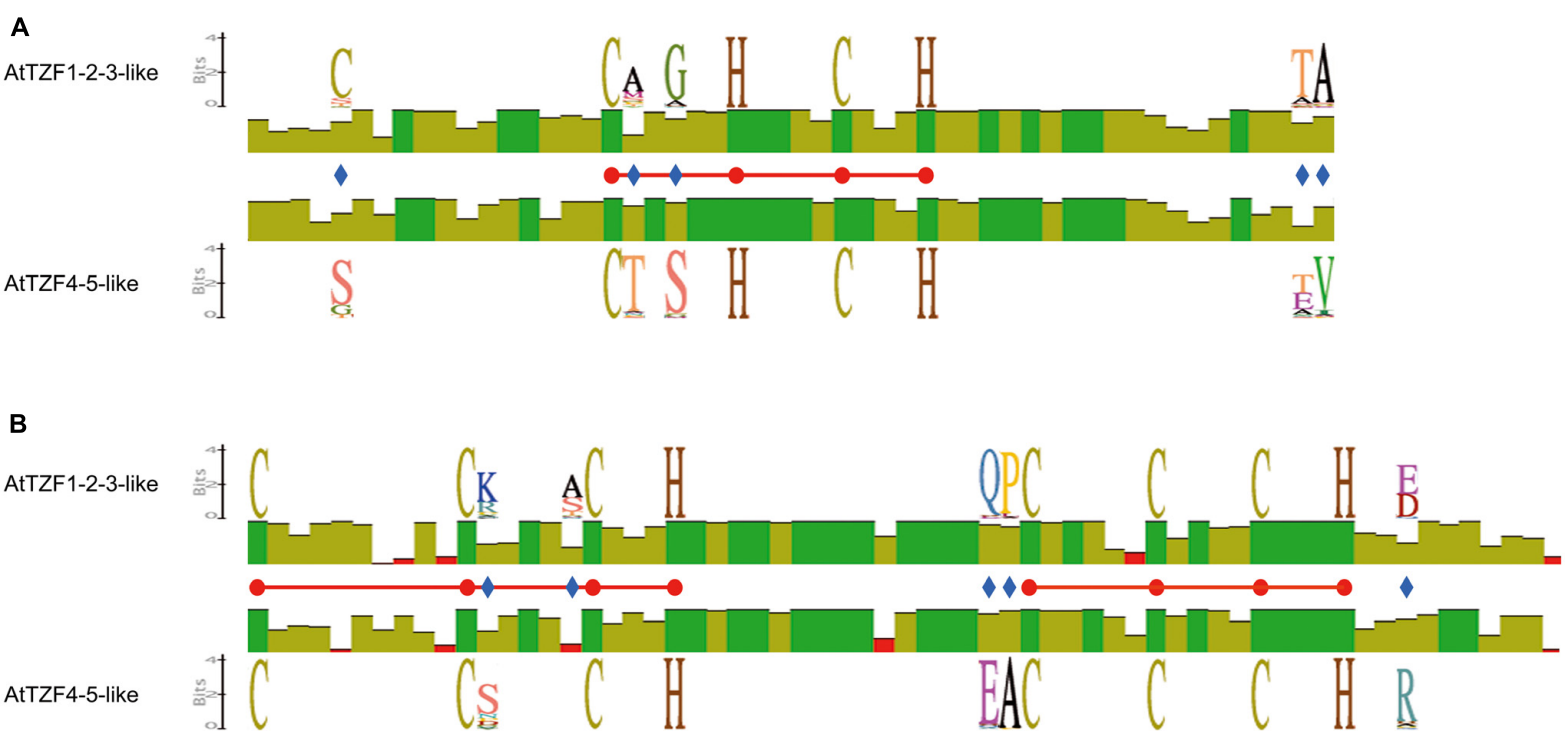
**Schematic representation of the conserved amino acids in the AtTZF1-2-3-like and AtTZF4-5-like proteins.** The scheme shows identity levels (histograms) at each amino-acid position, the sequence logo of invariant but specific positions (blue diamonds) in the AtTZF1-2-3-like and the AtTZF4-5-like proteins and the CHCH motif **(A)** and CCCH domain **(B)** amino acids (red balls, horizontal bars). Data were extracted from multi-alignments of the AtTZF1-2-3-like or AtTZF4-5-like full-length amino-acid sequences, constructed using the Clustal W algorithm with the Geneious software (version 5.5.3).

To investigate the conservation and divergence of the RR-TZF IIa and RR-TZF IIb protein subgroups, we constructed relative multiple sequence alignments and carried out comparisons. Within the RR-TZF region, the same conserved amino-acid positions seen in **Figure 1** were observed, which confirmed that these amino acids are specific for the respective groups and are evolutionarily conserved; the frequencies of the amino acids at each position are detailed in Supplementary Table S6. Among the conserved amino-acid residues, we focused our attention on the Cys (**Figure 6**, blue diamond) at position -12 from the first Cys of the CHCH motif. Most of the RR-TZF IIa proteins (84%) have this additional Cys. With the exception of a few cases, i.e., some angiosperms (e.g., *T. urartu*, *A. halleri*, *Carica papaya*, *Ricinus communis*) and all of the algal proteins, the proteins with this additional Cys were all in species that are further along the evolutionary scale.

These protein alignments also showed that some small regions that are up-stream and down-stream of the RR-TZF motif are conserved (Supplementary Figures S4 and S5). In the N-terminal region, there was the conservation of an IPP motif, which in the RR-TZF IIb proteins was followed by RKLL, and in the RR-TZF IIa proteins, by W. This W residue is present only in angiosperms, monocotyledons and dicotyledons, and not in other species (data not shown). In addition, the AtTZF1-2-3-like proteins contained another conserved short motif, RYLP, which is not found in the AtTZF4-5-like proteins. In the C-terminal region, the AtTZF1-2-3-like and AtTZF4-5-like proteins share some common motifs, such as SP-rich regions, and the sequence PDVGWVSELV/DPDLGWVNDLL, which is highly conserved. Finally, an EE—PAMER-VESGRDLR motif is evolutionarily conserved in the RR-TZF IIa proteins (Supplementary Figure S4), while there is a CCLFC motif in the RR-TZF IIb proteins (Supplementary Figure S5).

### Phylogenetic Analysis of the RR-TZF Group II Proteins

**FIGURE 7:**
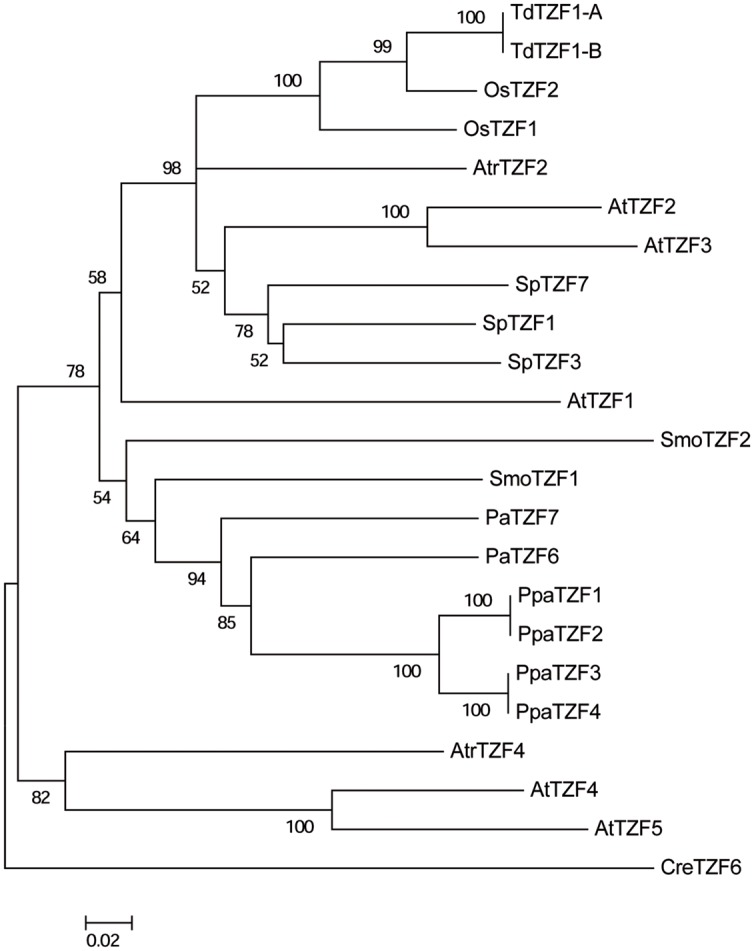
**Phylogenetic tree of the AtTZF1-2-3-4-5-like proteins from selected species.** The unrooted tree was constructed using the neighbor-joining method after alignment of the full-length amino-acid sequences using the Clustal W algorithm. Bootstrap values from 1,000 replicates are indicated at each node, and only bootstrap values higher than 50% from 1,000 replicates are shown. Scale bar: estimated 0.2 amino-acid substitutions per site.

To reveal the evolutionary relationships of the RR-TZF group II proteins, a neighbor-joining tree was constructed based on the alignment of the full-length amino-acid sequences of some selected plant species that are representative of each level of the evolutionary scale: *A. thaliana, O. sativa, T. durum, Spirodela polyrhiza, A. trichopoda, P. abies, Selaginella moellendorffii, P. patens*, and *C. reinhardtii* (**Figure 7**).

The CreTZF6 protein is located on the outer branch of the tree, which highlights the great phylogenetic distance between the algae and the land species. The other sequences can be divided into two groups, one that includes AtTZF4-5 and their *A. trichopoda* ortholog (AtrTZF4). As already indicated in the previous analysis, the AtTZF4-5 proteins appear not to have any orthologs in either monocotyledons or in non-angiosperm species, which indicates their recent origin and lineage specificity in dicotyledons. The presence of AtTZF4-5 in *A. trichopoda* suggests that this gene already existed in the ancestor of the angiosperms, but only in the dicotyledons has it been maintained, while in the monocotyledons it has been lost. The other group consists of 19 proteins, and it includes the AtTZF1-2-3 proteins and their relative orthologs that are present in all of the species analyzed, with the exception of algae. Moreover, clear separation is evident (which is statistically well supported) between the angiosperm and non-angiosperm (i.e., gymnosperm, bryophyte) proteins. Within the angiosperm group, the *T. durum* sequences are more similar to the *O. sativa* proteins, which is consistent with the evolutionary relationship between these species. Moreover, it is of note that despite it being a monocotyledon, the sequences of *S. polyrhiza* are closer to those of *Arabidopsis*, as compared to other monocotyledons. Within the non-angiosperm group, all of the *P. patens* sequences are highly related, so as to form a specific and well-supported group. The other sequences (e.g., *S. moellendorffii*, *P. abies*) are not distributed in a manner consistent with their phylogenetic relationships, which suggests that lycophytes and gymnosperms have undergone distinct and separate evolution.

### Expression Profiles of *RR-TZF Physcomitrella* Genes under Salt-Stress Conditions

**FIGURE 8:**
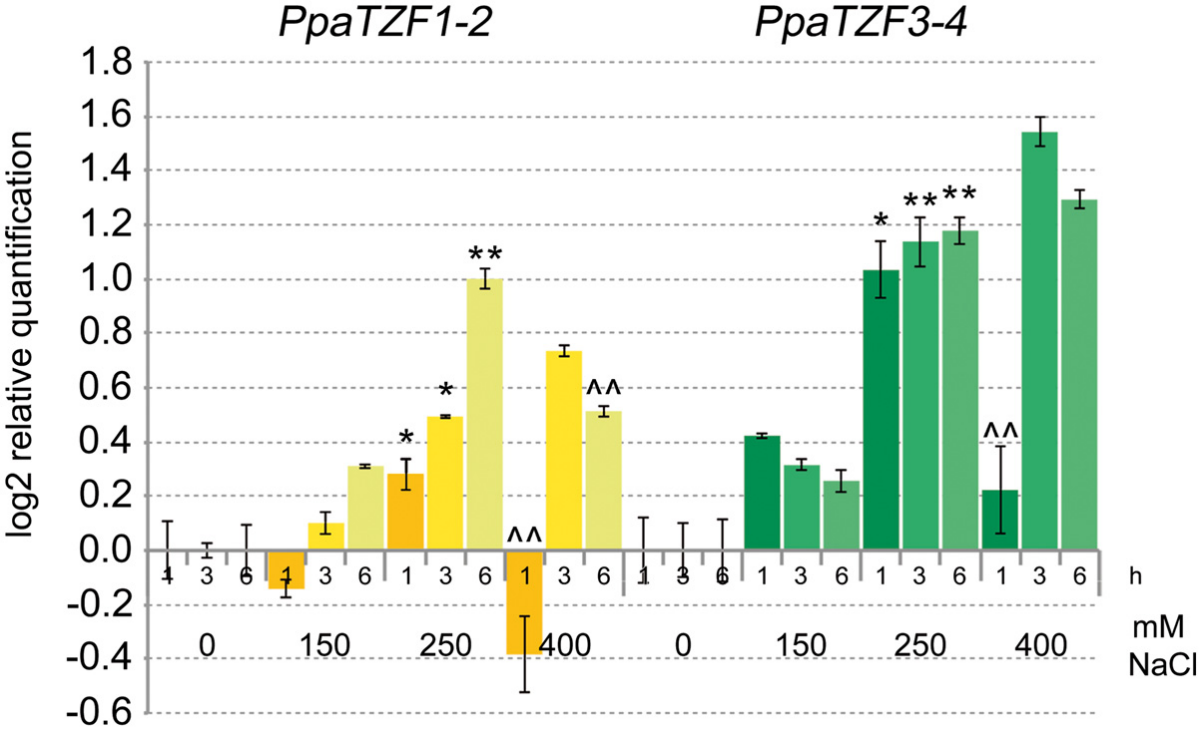
**Expression analysis of the *PpaTZF* genes under salt-stress conditions.** Protonemata were grown for 6 days on control medium, and then either transferred onto the same medium or onto medium containing 150, 250, or 400 mM NaCl, for different times. The relative expression levels are shown for *PpaTZF1-2* and *PpaTZF3-4* under control conditions and upon exposure to salt stress for 1, 3, and 6 h. The relative mRNA expression levels of each salt-treated sample were referred to the corresponding time point of the control experiment (no NaCl). Data are means of relative quantification (Log2) of two biological replicates normalized to *PpaEF1a* (±SE). Statistical significance was assessed by one-way ANOVA analysis followed by Bonferroni’s tests. °*P* < 0.05, ^∘∘^*P* < 0.01 1 h 150 mM vs 1 h 0 mM, 3 h 150 mM vs 3 h 0 mM, 6 h 150 mM vs 6 h 0 mM; ^∗^*P* < 0.05, ^∗∗^*P* < 0.01 1 h 250 mM vs 1 h 150 mM, 3 h 250 mM vs 3 h 150 mM, 6 h 250 mM vs 6 h 150 mM; ^∧^*P* < 0.05, ^∧∧^*P* < 0.01 1 h 400 mM vs 1 h 250 mM, 3 h 400 mM vs 3 h 250 mM, 6 h 400 mM vs 6 h 250 mM.

Previous studies have shown that *P. patens* can survive severe dehydration, high salinity, low temperature, and high osmotic stress (Frank et al., 2005; Saavedra et al., 2006; Charron and Quatrano, 2009). To investigate whether the *RR-TZF Physcomitrella* genes have conserved responses to salt stress, we analyzed their expression profiles in response to NaCl. The *PpaTZF1* and *PpaTZF2* genes are identical, and the *PpaTZF3* and *PpaTZF4* genes are nearly identical. Therefore, only two distinct assays for the qPCR analysis were developed here, one that amplified the *PpaTZF1-2* pair, and the other that amplified the *PpaTZF3-4* pair. In analogy with the experiments performed in *Arabidopsis* and durum wheat (see **Figure 3**), the *Physcomitrella* protonemata were treated with different concentrations of NaCl (i.e., 0, 150, 250, 400 mM) for 1, 3, and 6 h (**Figure 8**). Very interestingly, both pairs of *Physcomitrella* genes showed clear dose-dependent up-regulation in response to the salt. At the lowest NaCl concentration (150 mM), *PpaTZF1-2* was slightly induced 6 h after the salt exposure. At 250 mM NaCl, the *PpaTZF1-2* transcript levels were about 1.4-fold higher than those observed under control conditions 3 h after this salt exposure. At the highest salt concentration (400 mM NaCl), induction of the *PpaTZF1-2* genes was transient, with the maximum transcript levels reached after 3 h of salt treatment (about 1.7-fold induction). Similar behavior was shown by the *PpaTZF3-4* pair, which overall appeared to be slightly more inducible than the *PpaTZF1-2* pair.

In conclusion, the expression profiles here indicated clear responses to salt for the *RR-TZF Physcomitrella* genes, which were similar to those of the durum wheat *TdTZF1-A* and *TdTZF1-B* genes, and of the *Arabidopsis* group II *RR-TZF* genes. This thus suggests a conserved function of these proteins across these evolutionarily distant plant organisms.

## Discussion

A number of *RR-TZF* genes have been identified previously in higher plants, and their products have been shown to have important roles in the regulation of some developmental processes and adaptive responses to abiotic stress, such as to cold, salt and drought (Wang et al., 2008; Chai et al., 2012; Lee et al., 2012). In the present study, we characterized two durum wheat *RR-TZF* genes, *TdTZF1-A*, and *TdTZF1-B*, which are highly homologous to *AtTZF2* and *AtTZF3*. Using a short conserved peptide sequence derived from the RR-region, we identified 461 putative RR-TZF plant proteins that share the unique signature of KX_3_CX_5_HX_4_CX_3_HX_6_RRX_6_YX_4_CX_7-8_CX_5_CX_3_HX_2_FEX_3_HPX_7_CX_5_CX_4_CFFAH, which includes a highly conserved zinc-finger CCCH domain. Remarkably, *RR-TZF* genes were found in all levels of the evolutionary scale, including the green algae *Coccomyxa subellipsoidea C-169*, indicating the evolutionary origin of these genes in a common ancestor of green algae and land plants. Based on our homology analysis, we also divided the RR-TFZ family of proteins into five different groups, as RR-TZF I-V, and TdTZF1-A and TdTZF1-B belong to the second of these groups, RR-TZF II.

### The RR-TZF II Group

The RR-TZF II group is formed by 168 proteins that are encoded by all of the plant genomes sequenced to date, with the only exception of some green algae. This suggests that the evolutionary origin of the RR-TZF II group is also very ancient, and is likely to have been before the divergence of the algae and the land plants. However, the homology analysis indicated that this group can be further divided in two subgroups: RR-TZF IIa and RR-TZF IIb. The first of these subgroups includes the AtTZF1-3-like proteins, and the second subgroup includes proteins that are homologous to AtTZF4/SOM and AtTZF5. Most of the RR-TZF IIa proteins (84%), including the TdTZF1-A, TdTZF1-B, AtTZF2-3, and *P. patens* proteins, have an additional Cys amino acid that is nine residues upstream of the invariant Lys of the RR-TZF signature (see **Figure 6**). The conservation of this amino-acid residue suggests that it might have a specific role in protein folding and/or protein-protein interactions; e.g., through disulfide bonding. As an alternative, this additional Cys might be involved in the formation of an atypical CCCH domain, as C-X_12_-C-X_10_-C-X_3_-H, which partially overlaps with the CHCH motif, as suggested by Huang et al. (2011).

### Evolutionarily Conserved Regulation for the RR-TZF IIa Proteins

We and others have shown that several genes belonging to the RR-TZF II group, which include the *P. patens* genes described here, respond to salt stress (Sun et al., 2007; Jan et al., 2013; Han et al., 2014; Wang et al., 2014; Zhou et al., 2014). The conservation of the gene expression response to salt stress suggests a function for the RR-TZF II proteins in assisting plants to cope with environmental stress, a common challenge to phylogenetically distant plant species. Interestingly, we observed very similar expression patterns for the *Arabidopsis* (i.e., *AtTZF2*, *AtTZF3*) and durum wheat (i.e., *TdTZF1-A*, *TdTZF1-B*) genes during the germination process in the presence of NaCl. The conservation of salt-stress regulation during germination is intriguing considering the physiological diversity of the process across these two species, due to the different morpho-physiological features of *Arabidopsis* and durum wheat seeds. Remarkably, the attenuation and overexpression of *AtTZF3* in plants showed altered germination ability exclusively in the presence of NaCl and ABA, which thus suggests that the activity of the AtTZF3 protein is dependent on salt-induced stress and/or on the ABA levels. Previous studies have provided evidence for roles of the other *Arabidopsis RR-TZF II* genes in the germination process; however, in those cases, the germination appeared to correlate inversely to the level of expression of the selected genes. For instance, the concurrent suppression of *AtTZF1-3* by RNAi was characterized by early germination and relatively stress-sensitive phenotypes. Also, rice plants overexpressing *OsTZF1* showed delayed seed germination and growth delay at the seedling stage, whereas RNAi knock-down *OsTZF1* lines showed early seed germination and enhanced seedling growth (Jan et al., 2013). Similarly, knock-out mutants of *AtTZF4/SOM*, *AtTZF5*, or *AtTZF6* showed early germination, whereas the plants overexpressing these genes showed late germination, compared to the wild-type (Bogamuwa and Jang, 2013).

A role for the RR-TZF II proteins in salt-stress responses might have arisen very early during plant evolution. Later, in higher plants, the RR-TZF II proteins might have acquired new regulatory functions that are connected to the germination process. However, ABA represents a common factor that links together salt stress and germination. ABA has an ancient origin and conserved functions in the life of plants, as it affects growth and differentiation, increases dehydration stress tolerance in bryophytes, and induces accumulation of soluble sugars (such as compatible solutes) in association with enhancement of freezing tolerance in *P. patens* protonemata (Nagao et al., 2005, 2006). The core network that mediates ABA signaling is evolutionarily conserved between *P. patens* and angiosperms (Takezawa et al., 2011), and the mechanisms of regulation of ABA-mediated salt-stress responses are very similar in *P. patens* and in seed plants (Richardt et al., 2010). In particular, Richardt et al. (2010) demonstrated that the *P. patens* homologs of *Arabidopsis* genes have key roles in the regulation of processes like flowering and seed development. In light of these data, our results suggest that the regulation of *RR-TZF II* gene expression during germination might have evolved from the pre-existing pathway(s) that regulate ABA-mediated responses to salt stress. This hypothesis is also supported by the conservation of certain regulatory elements that are linked to the responses to ABA and to abiotic stress, and that are located within the promoters of the *AtTZF1-5* genes and their homologs in durum wheat and *P. patens* (data not shown). As an example, the ABRERATCAL element, which is an ABA-responsive element (Finkler et al., 2007), is present in the promoters of the *AtTZF1-4*, *TdTZF1-B*, and *P. patens RR-TZF* genes.

### Expansion the RR-TZF II Genes in Dicotyledons

The level of sequence similarity and the physical position of the *TdTZF1-A* and *TdTZF1-B* genes on chromosomes 3A and 3B, respectively, suggest that these two genes are homologous in the durum wheat genome. As *TdTZF1-A* and *TdTZF1-B* are characterized by similar expression profiles, this strongly suggests a conserved function for these two genes in the diploid progenitors of durum wheat that was retained after the polyploidization event, probably because of their importance in stress responses and seed germination. A similar explanation can be proposed for the *RR-TZF-IIa* genes in *Arabidopsis*, which have highly conserved expression profiles across duplicated genes. This is consistent with the ‘functional buffering’ hypothesis that stipulates that genes with crucial functions tend to be retained to ensure that the essential functions can be carried out in the event of inactivation of one of the duplicates (Chapman et al., 2006). In contrast, the *RR-TZF-IIb* group is lacking in monocots and non-angiosperm species, which indicates a recent origin and lineage specificity in dicot plants. However, the occurrence of one *RR-TZF-IIb* gene in *A. trichopoda*, which is considered to be the most primitive angiosperm plant, suggests that genes related to the *RR-TZF-IIb* group might have been lost during the evolution of monocot plants.

Among these *RR-TZF-IIb* genes there is *AtTZF4*/*SOM*, for which a role as a negative regulator of phytochrome-dependent seed germination has been established. Although *Arabidopsis* shares some dormancy pathway components with cereals (e.g., ABI3, VP1), there are also major differences in terms of the responses to light, as well as to other environmental cues (Simpson, 1990; Goggin et al., 2008; Gubler et al., 2008). Interestingly, however, a model for temperate cereals, *Brachipodium distachyon*, shows phytochrome regulation of seed dormancy and germination (Barrero et al., 2011), even though it lacks any of the *RR-TZF-IIb* genes.

Altogether, these data suggest that the expansion of the *RR-TZF-II* genes might have been related to the divergence between the monocot and dicot lineages, rather than to a specialized functional response to light stimuli. Among other possibilities, it appears reasonable to hypothesize that once the *AtTZF4* gene acquired phytochrome-mediated control, its product became a crucial factor in light-regulated germination, because of its intrinsic function as a negative regulator of this process, which is a feature that it shares with other members of the *RR-TZF-II* family.

## Conclusion

The present study provides a complete survey and classification of the *RR-TZF* genes in plants for which the genomic information is available. This analysis has allowed us to compile a detailed description of the structural features of the RR-TZF motifs in relation to the evolution of this gene family. The strong conservation in terms of their sequence and structural features that is shown here across phylogenetically distant species, together with the similar expression profiles of the *RR-TZF* genes in response to salt stress in *Arabidopsis*, durum wheat, and the moss *P. patens*, have allowed us to hypothesize that these genes emerged as key regulators of stress responses very early during plant evolution.

## Conflict of Interest Statement

The authors declare that the research was conducted in the absence of any commercial or financial relationships that could be construed as a potential conflict of interest.

## Footnotes

1 https://urgi.versailles.inra.fr/blast/blast.php

2 http://www.phytozome.net/

3 http://planttfdb.cbi.pku.edu.cn/

4 http://trifldb.psc.riken.jp/ver.2.0/blast.pl

5 http://plantta.jcvi.org/search.shtml

6 http://www.wheatgenome.org/content/view/full/625

7 http://blast.ncbi.nlm.nih.gov/Blast.cgi

8 http://bbc.botany.utoronto.ca/efp/cgi-bin/efpWeb.cgi

9 http://www.plexdb.org/index.php
