# Supplementary material for: Conservation of AtTZF1, AtTZF2, and AtTZF3 homolog gene regulation by salt stress in evolutionarily distant plant species
Source: Front Plant Sci. 2015 Jun 16;6:394. doi: 10.3389/fpls.2015.00394 (PMC4468379; doi:10.3389/fpls.2015.00394)
Supplement: Supplementary file 1 [file Supplementary_Materials.ZIP › correct files/132224_Morelli_Supplementary Materials.PDF]

Supplementary Materials

Conservation of *AtTZF1*, *AtTZF2* and *AtTZF3* homolog gene regulation in evolutionarily distant plant species

Fabio D'Orso[1†], Anna Maria De Leonardis[2,3†], Sergio Salvi[1], Agata Gadaleta[4], Ida Ruberti[5], Luigi Cattivelli[2,6], Giorgio Morelli[1*], Anna Maria Mastrangelo[2*]

[1]Food and Nutrition Research Centre, Council for Agricultural Research and Economics (CRA), Via Ardeatina 546, 00178 Roma (Italy)
[2]Cereal Research Centre, Council for Agricultural Research and Economics (CRA), SS 16 Km 675, 71122 Foggia (Italy)
[3]Department of the Sciences of Agricolture, Food and Environment, University of Foggia, Via Napoli 25, 71122 Foggia (Italy)
[4]Department of Soil, Plant and Food Sciences, "Aldo Moro" University of Bari, Via G. Amendola 165/A, 70126 Bari (Italy)
[5]Institute of Molecular Biology and Pathology, National Research Council (CNR), Piazzale Aldo Moro 5, 00185 Roma (Italy)
[6]Genomics Research Centre, Council for Agricultural Research and Economics (CRA), Via San Protaso 302, 29017 Fiorenzuola d'Arda (Italy)

orcid codes: (F.D.: 0000-0002-4906-0026; I.R.: 0000-0002-4974-5818; G.M.: 0000-0002-8994-4838.)

*** Correspondence:**
Anna Maria Mastrangelo, CRA-Cereal Research Centre, SS 16 Km 675, 71122 Foggia (Italy)
annamaria.mastrangelo@entecra.it
Giorgio Morelli, CRA-Food and Nutrition Research Centre, Via Ardeatina 546, 00178 Roma (Italy)
giorgio.morelli@entecra.it

† Authors equally contributing to the work

Supplementary Materials and Methods

1.1. TdTZF1-A/B amplifications

The amplification reactions for *TdTZF1-A* were performed under the following conditions: preheating at 94 °C for 5 min, then 32 cycles of denaturation at 94 °C for 60 s, annealing at 62 °C for 30 s, and extension at 72 °C for 2 min, followed by final extension for 7 min. Genomic DNA and cDNA were used as templates in two different PCR reactions.

The amplification reactions for *TdTZF1-B* were performed under the following conditions: preheating at 94 °C for 5 min, then 32 cycles of denaturation at 94 °C for 60 s, annealing at 58 °C for 30 s, and extension at 72 °C for 2 min, followed by final extension for 7 min. Genomic DNA and cDNA were used as templates in two different PCR reactions.

The primers used are detailed in Supplementary Table S1.

1.2. Arabidopsis cloning procedure

For overexpression of *AtTZF3*, the *AtTZF3* open-reading frame was PCR amplified from genomic DNA (for primer sequences, see Supplementary Table S1). The PCR reactions were performed on an Applied Biosystems 2720 thermocycler (Life Technologies) using Phusion®High-Fidelity DNA Polymerase kits (Finnzymes). The amplification reactions were carried out using 0.2 mM dNTPs, Phusion® HF Buffer 1×, 0.5 μM each primer, and 0.02 U/μl Phusion® DNA Polymerase, in a volume of 40 μl. The PCR reaction conditions were set as follows: 30 s at 98 °C for initial DNA denaturation, 30 cycles of DNA amplification for 10 s at 98 °C (denaturation), 30 s at 60 °C (for primers annealing), 30 s at 72 °C (for DNA extension), and 5 min at 72 °C for the final extension. The final PCR product was gel-purified and inserted into pENTR/D-TOPO (Life Technologies). Next, the *AtTZF3* open-reading frame was recombined into the 2× [^{35}S] expression vector pMDC32 (Curtis and Grossniklaus, 2003).

The *AtTZF3* amiRNA construct was designed using a web-based program (http://wmd2.weigelworld.org) (Ossowski et al., 2008, Schwab et al., 2006). The pRS300 plasmid was used as the template to create the amiRNA (Ossowski et al., 2008). The primer sequences were as detailed in Supplementary Table S1. The mA and mB primers are different with respect to those indicated by Schwab et al. (2006), and they also contain Gateway adaptor sequences for recombination reactions. The final PCR product was gel-purified and inserted into pDONR221. Next, it was recombined into the pBENDER plasmid.

For the ihpRNA construct, the *AtTZF3* target sequence (region from nucleotide 18 to 136, starting from first nucleotide of the coding sequence) was PCR amplified from genomic DNA using the primers reported in Supplementary Table S1. The amplified PCR product was gel-purified and cloned into pENTR/D-TOPO (Life Technologies), and subsequently into the pK7GWIWG2 (II) RNAi vector via recombination reactions.

After cloning in *Escherichia coli* DH5a cells, all of the constructs were sequenced and subsequently transferred to *Agrobacterium tumefaciens* cells (strain GV3101) that harbored the pMP90RK (for overexpression and ihpRNA constructs) or pMB90RK (for amiRNA construct) plasmids.

After transformation, the transgenic plants were selected on half-strength MS medium that contained the appropriate antibiotics. Transgenic lines segregating 3:1 for antibiotic resistance were selected in the T_2 generation of each transformation, and the T_3 homozygous generation was used for the subsequent analysis.

Supplementary Figures

Supplementary Figure S1. Physical mapping of the *TdTZF1-A* and *TdTZF1-B* genes to chromosomes 3A and 3B of wheat. Fragments specific for each gene were amplified in nulli-tetrasomic (N3AT3D, N3BT3D, N3DT3B), ditelosomic (DT3AS, DT3AL, DT3BS, DT3BL) and deletion lines (3AS4-0.45, 3AS2-0.23, 3AL3-0.42, 3AL5-0.78, 3BS8-0.78, 3BS9-0.57, 3BS1-0.33, 3BL7-0.63) for chromosomes of group 3 of the durum wheat cv. 'Chinese Spring'. The amplification

products were separated on agarose gels and are represented with the 3A and 3B deletion bin maps of chromosomes 3A and 3B, as described by Gadaleta et al. (2009).

Supplementary Figure S2. *In-silico* expression analysis of the Arabidopsis and wheat *RR-TZF* genes. (A) Expression profile of the Arabidopsis *RR-TZF* genes during development, based on the data of the developmental map of the Arabidopsis eFP Browser (http://bbc.botany.utoronto.ca/efp/cgi-bin/efpWeb.cgi). (B) Expression profile of the Arabidopsis *RR-TZF* genes under abiotic stress conditions, based on the data of the Arabidopsis eFP Browser (http://bbc.botany.utoronto.ca/efp/cgi-bin/efpWeb.cgi). (C) Expression profile of wheat *RR-TZF* genes during development, based on the data of experiment TA3: Transcription patterns during wheat development, at the PLEXdb database (http://www.plexdb.org/modules/PD_browse/experiment_browser.php?experiment=TA3). It should be noted that *TaTZF1/TdTZF1* and *TaTZF5* were induced in 22 DAP embryos and endosperms, in comparison to 3-5 DAP caryopsis. (D) Expression profile of wheat *RR-TZF* genes under drought conditions, based on the data of experiment TA23: Drought stress in wheat at grain filling stage, at PLEXdb database (http://www.plexdb.org/modules/PD_browse/experiment_browser.php?experiment=TA23). Here, it should be noted that the *TaTZF1/TdTZF1* gene shows a repression under severe drought stress in 'Creso' and 'Chinese Spring' 5AL deletion line (CS-5AL) compared to 'Chinese Spring'. (E) Expression profile of wheat *RR-TZF* genes under cold conditions, based on the data of experiment TA22: Freeze Resistance basis of winter wheat mutant lines, at the PLEXdb database (http://www.plexdb.org/modules/PD_browse/experiment_browser.php?experiment=TA22). During cold stress, *TaTZF1*, *TdTZF1* and *TaTZF5* showed two-fold increases in mRNA levels compared 4°C to 25 °C for both genotypes, SD169029 and SD16169.

Supplementary Figure S3. RR-TZF protein family conservation in the plant kingdom. (A) Dendrogram of all of the RR-TZF proteins from the species investigated along the evolutionary scale, as constructed using the Geneious software (version 5.5.3) with the neighbor-joining method. The sequences can be divided into five major groups, which are indicated as I to V. (B) Multi-alignment of all of the RR-TZF full-length amino-acid sequences, as constructed using the Clustal W algorithm with the Geneious software (version 5.5.3). The sequence Logo, consensus sequence, and identity level of the RR and TZF regions are shown. Alignment was manually edited by adjusting the spacing between the first and second Cys of the CCCH domains, highlighting the conventional spacings C-X_{7-8}-C-X_5-C-X_3-H and C-X_5-C-X_4-C-X_3-H for the first and second CCCH domains, respectively. Different spacings represent structural features of a few sequences. The CHCH motif and the CCCH domains are highlighted in red; invariant amino-acid positions in all of the RR-TZF sequences are represented by blue balls. Overall, the conserved amino acids provide a specific signature.

Supplementary Figure S4. Conservation of the AtTZF1-2-3-like proteins. Multi-alignment of AtTZF1-2-3-like full-length amino-acid sequences, as constructed using the Clustal W algorithm with the Geneious software (version 5.5.3), with the consensus sequence, sequence logo and identity levels shown. Conserved motifs specific to the AtTZF1-2-3-like proteins are highlighted in the violet boxes; conserved motifs shared with the AtTZF4-5-like proteins are indicated in the green boxes.

Supplementary Figure S5. Conservation of the AtTZF4-5-like proteins. Multi-alignment of the AtTZF4-5-like full-length amino-acid sequences, as constructed using the Clustal W algorithm with the Geneious software (version 5.5.3), with the consensus sequence, sequence logo and identity levels shown. Conserved motifs specific to the AtTZF4-5-like proteins are shown in the violet boxes; conserved motifs shared with the AtTZF1-2-3-like proteins are indicated in the green boxes.

References

Gadaleta, A., Giancaspro, A., Giove, S. L., Zacheo, S., Mangini, G., Simeone, R., Signorile, A. & Blanco, A. 2009. Genetic and physical mapping of new EST-derived SSRs on the A and B genome chromosomes of wheat. *Theor Appl Genet,* 118**,** 1015-25. doi: 10.1007/s00122-008-0958-1

Ossowski, S., Schwab, R. & Weigel, D. 2008. Gene silencing in plants using artificial microRNAs and other small RNAs. *Plant Journal,* 53**,** 674-690. doi: 10.1111/j.1365-313X.2007.03328.x

Schwab, R., Ossowski, S., Riester, M., Warthmann, N. & Weigel, D. 2006. Highly specific gene silencing by artificial microRNAs in Arabidopsis. *Plant Cell,* 18**,** 1121-33. doi: 10.1105/tpc.105.039834.
