# Supplementary material for: Conservation of AtTZF1, AtTZF2, and AtTZF3 homolog gene regulation by salt stress in evolutionarily distant plant species
Source: Front Plant Sci. 2015 Jun 16;6:394. doi: 10.3389/fpls.2015.00394 (PMC4468379; doi:10.3389/fpls.2015.00394)
Supplement: Supplementary file 1 [file Supplementary_Materials.ZIP › correct files/132224_Morelli_Supplementary_Figure_S2.PPTX]

## Slide 1
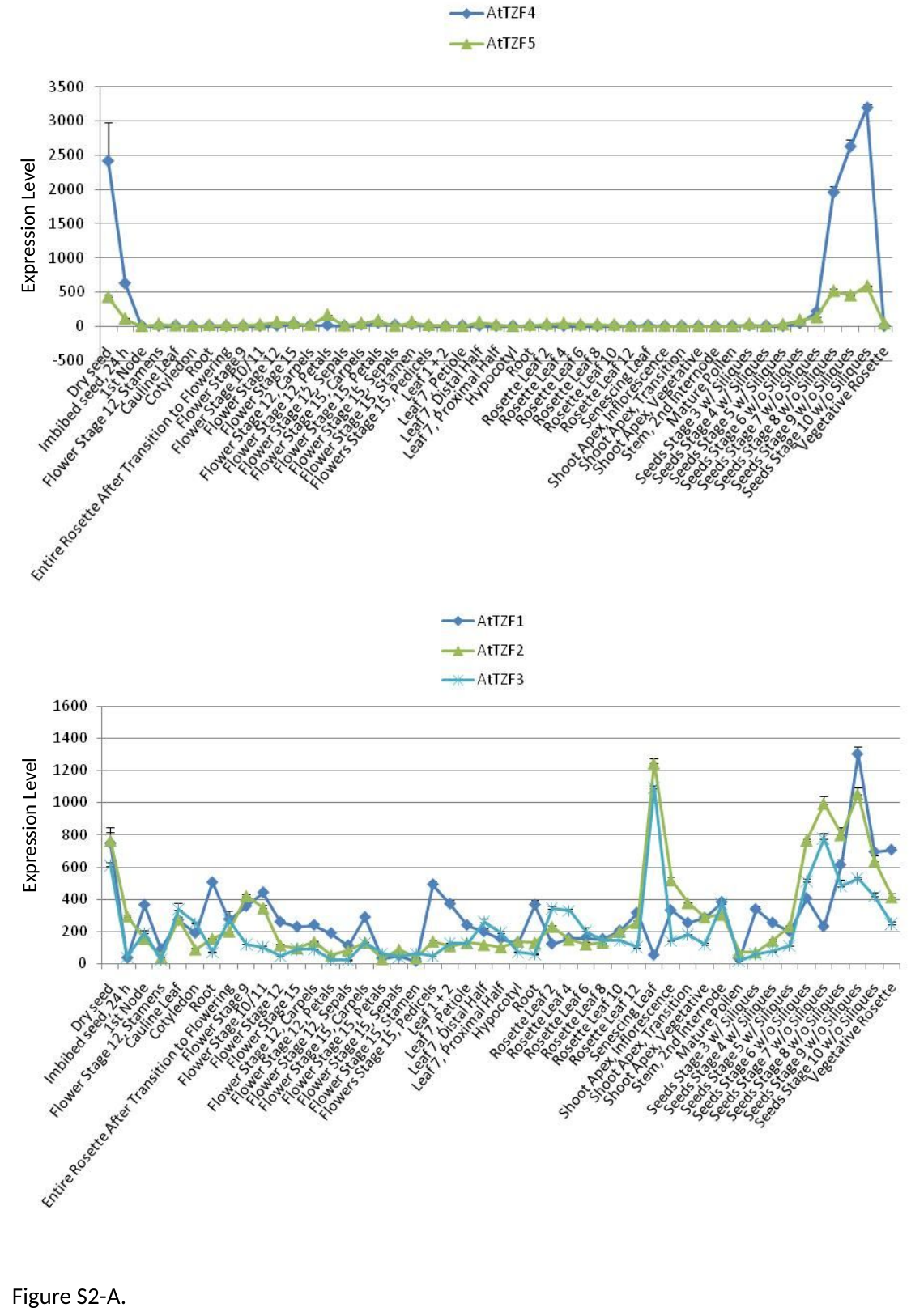

Expression Level
Expression Level
Figure S2-A.

## Slide 2
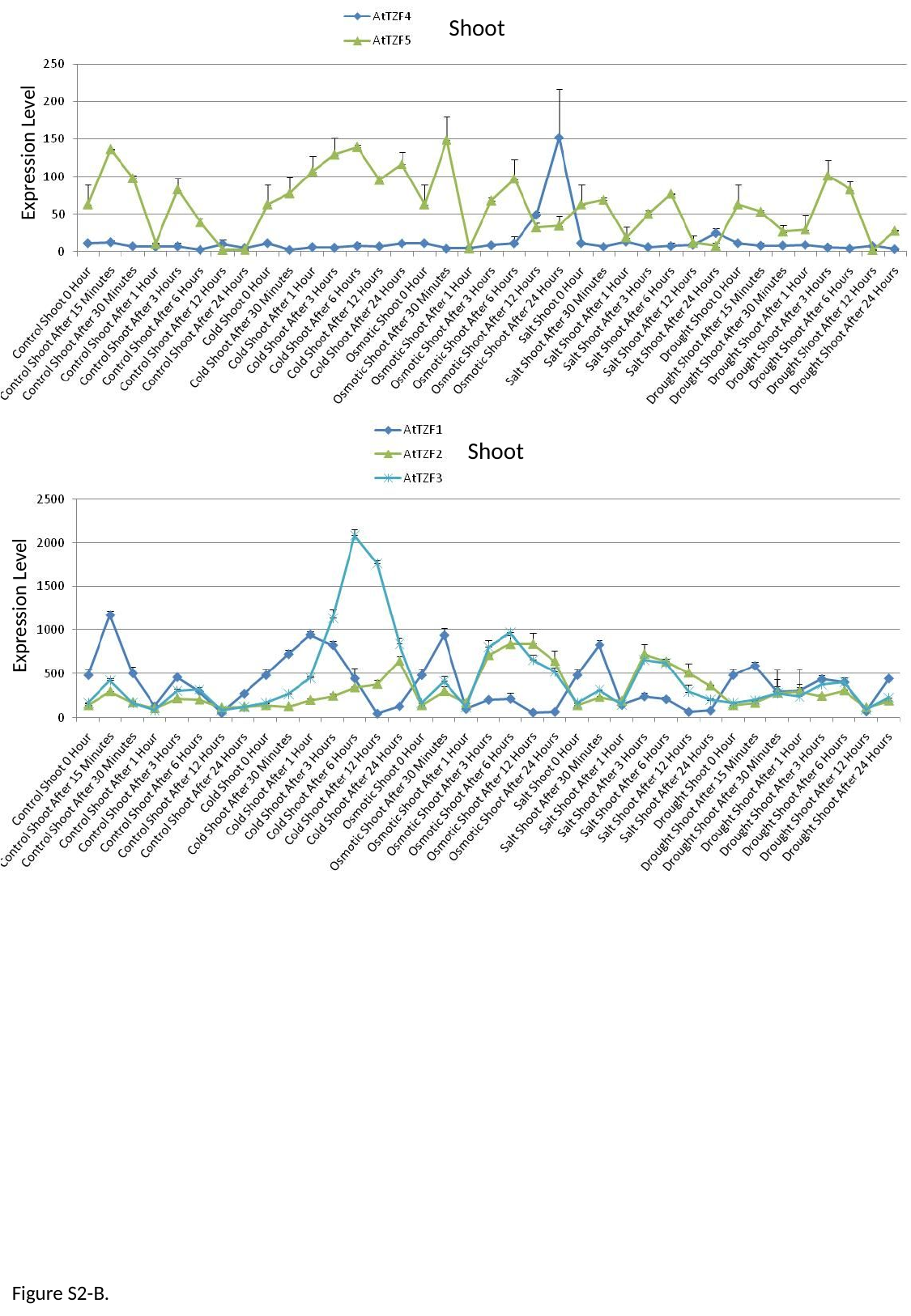

Shoot
Expression Level
Shoot
Expression Level
Figure S2-B.

## Slide 3
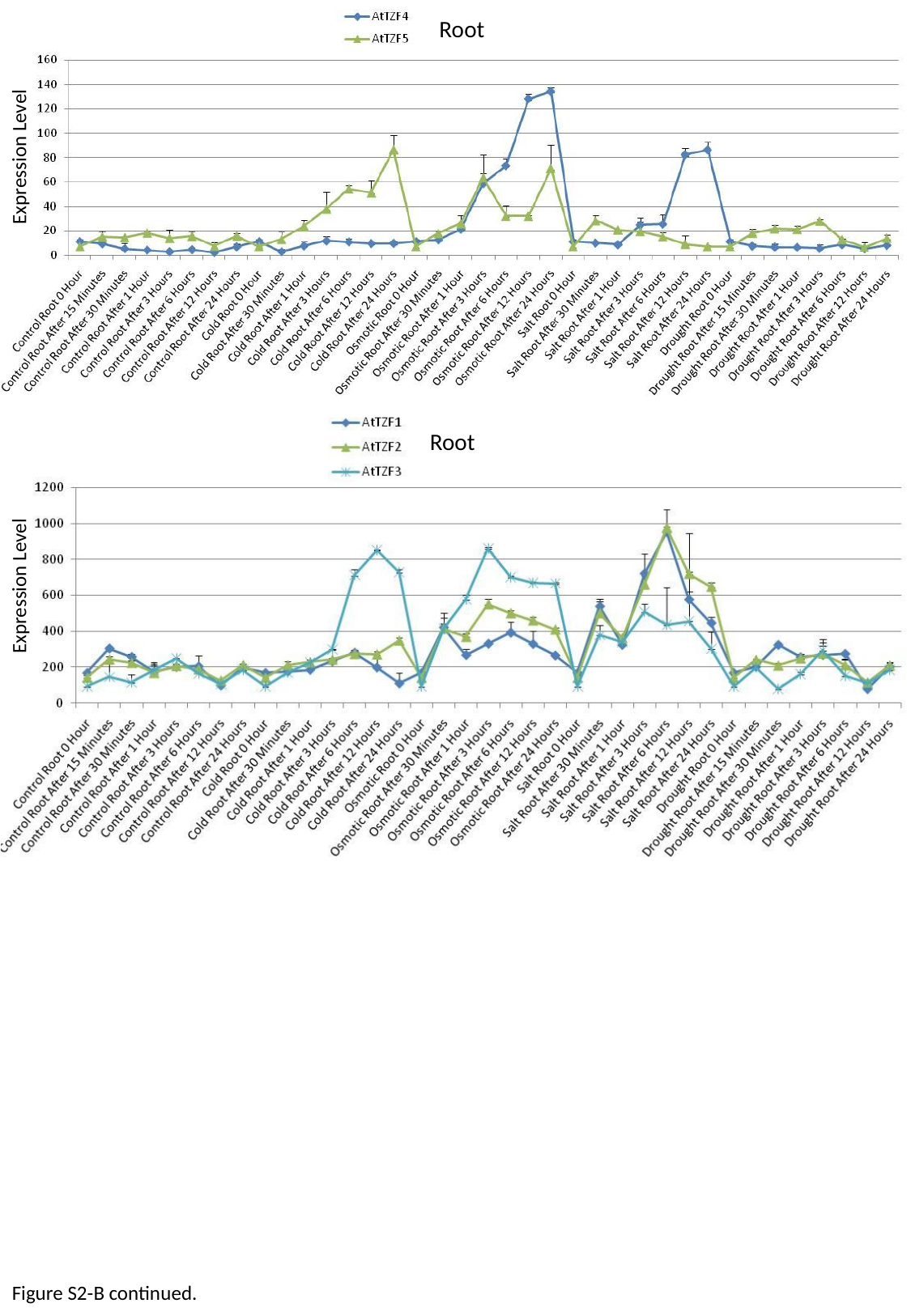

Root
Expression Level
Root
Expression Level
Figure S2-B continued.

## Slide 4
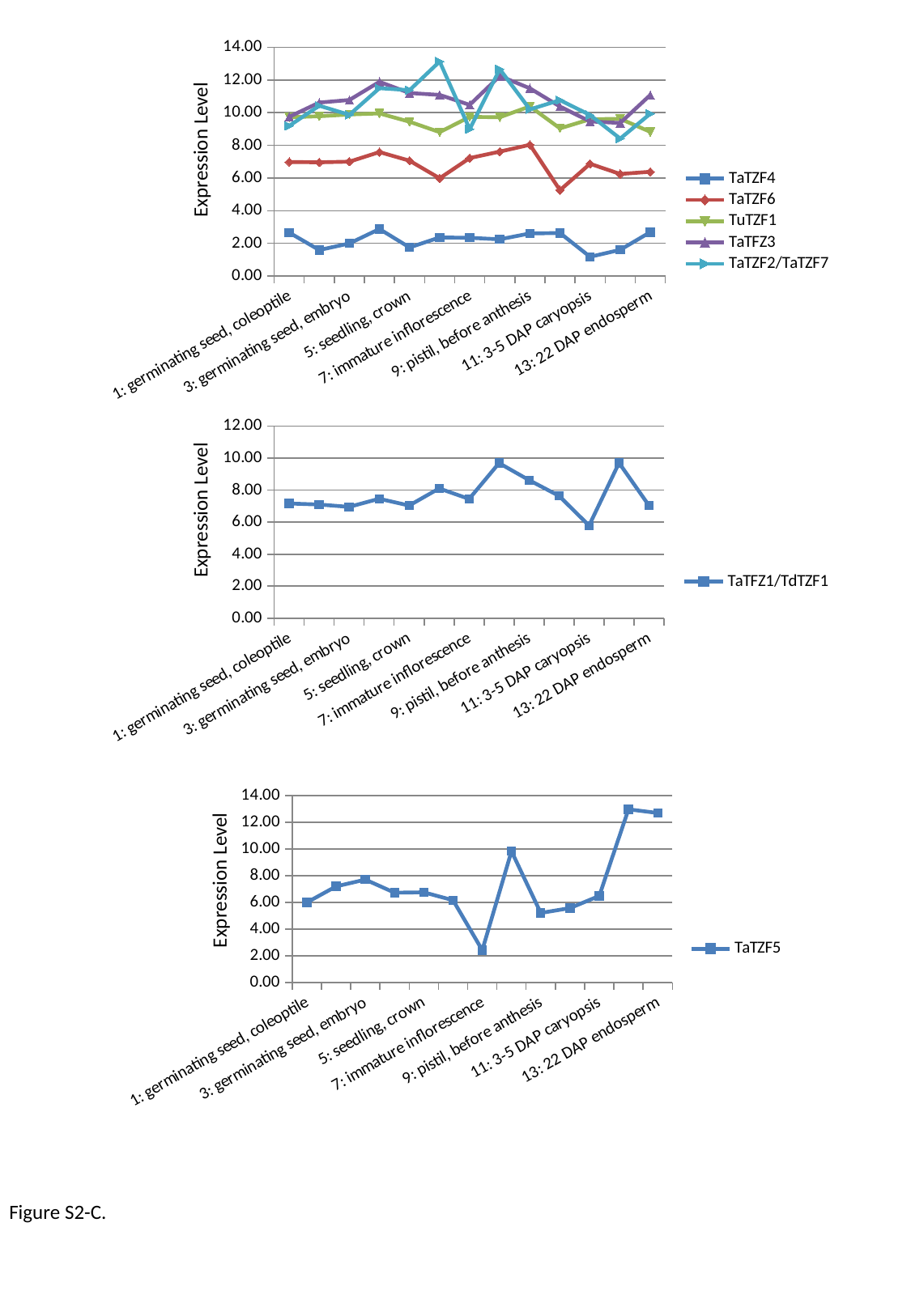

### Chart
| Category | TaTZF4 | TaTZF6 | TuTZF1 | TaTFZ3 | TaTZF2/TaTZF7 |
|---|---|---|---|---|---|
| 1: germinating seed, coleoptile | 2.6586308795203 | 6.9830958166038 | 9.721444583300865 | 9.74658798423058 | 9.194892077739501 |
| 2: germinating seed, root | 1.591488951453001 | 6.960576468467925 | 9.783320923069398 | 10.61826036797299 | 10.42656879697467 |
| 3: germinating seed, embryo | 1.993460317326066 | 7.001976533651368 | 9.88928080361265 | 10.77766629174001 | 9.869718197518067 |
| 4: seedling, root | 2.877298057438833 | 7.5887741421074 | 9.956216393131035 | 11.88835140508433 | 11.50060546658267 |
| 5: seedling, crown | 1.7480937256398 | 7.06257820969019 | 9.447253376751299 | 11.20469657645868 | 11.37845671059335 |
| 6: seedling, leaf | 2.360587377403635 | 5.985768815000843 | 8.806840406868805 | 11.08651782717435 | 13.13315146501765 |
| 7: immature inflorescence | 2.339678788128662 | 7.221944543625167 | 9.74325778682198 | 10.478677076815 | 8.99357577595186 |
| 8: floral bracts, before anthesis | 2.246780054435168 | 7.61644253549497 | 9.721570003140789 | 12.246813593807 | 12.66848876568367 |
| 9: pistil, before anthesis | 2.60400036049 | 8.037157795045065 | 10.386176724763 | 11.49075150114534 | 10.20102838925548 |
| 10: anthers, before anthesis | 2.630655735167 | 5.255089804608766 | 9.0382035390816 | 10.37695191885501 | 10.769860707955 |
| 11: 3-5 DAP caryopsis | 1.175608440484646 | 6.8724884687341 | 9.597168361588887 | 9.463814135459405 | 9.85532263803205 |
| 12: 22 DAP embryo | 1.602007330172038 | 6.24781535462103 | 9.623255231389132 | 9.37079723170418 | 8.41458423487282 |
| 13: 22 DAP endosperm | 2.683637267048471 | 6.3816322639624 | 8.82907957220962 | 11.08192545236333 | 9.937215951544202 |Expression Level
### Chart
| Category | TaTFZ1/TdTZF1 |
|---|---|
| 1: germinating seed, coleoptile | 7.162959420385784 |
| 2: germinating seed, root | 7.098544407111366 |
| 3: germinating seed, embryo | 6.95260752165277 |
| 4: seedling, root | 7.459458271655634 |
| 5: seedling, crown | 7.035548622394072 |
| 6: seedling, leaf | 8.102941521063633 |
| 7: immature inflorescence | 7.44717742258 |
| 8: floral bracts, before anthesis | 9.684182440377498 |
| 9: pistil, before anthesis | 8.62121182563286 |
| 10: anthers, before anthesis | 7.636355530136726 |
| 11: 3-5 DAP caryopsis | 5.786438550557967 |
| 12: 22 DAP embryo | 9.694572123028967 |
| 13: 22 DAP endosperm | 7.028229263011533 |Expression Level
### Chart
| Category | TaTZF5 |
|---|---|
| 1: germinating seed, coleoptile | 6.003769919090873 |
| 2: germinating seed, root | 7.21077018506241 |
| 3: germinating seed, embryo | 7.719583789924341 |
| 4: seedling, root | 6.73750381294377 |
| 5: seedling, crown | 6.766241277976698 |
| 6: seedling, leaf | 6.171157376566128 |
| 7: immature inflorescence | 2.424700032181734 |
| 8: floral bracts, before anthesis | 9.841777699002165 |
| 9: pistil, before anthesis | 5.217423517435027 |
| 10: anthers, before anthesis | 5.581495318785066 |
| 11: 3-5 DAP caryopsis | 6.491401085452188 |
| 12: 22 DAP embryo | 12.98507353416035 |
| 13: 22 DAP endosperm | 12.71052139924333 |Expression Level
Figure S2-C.

## Slide 5
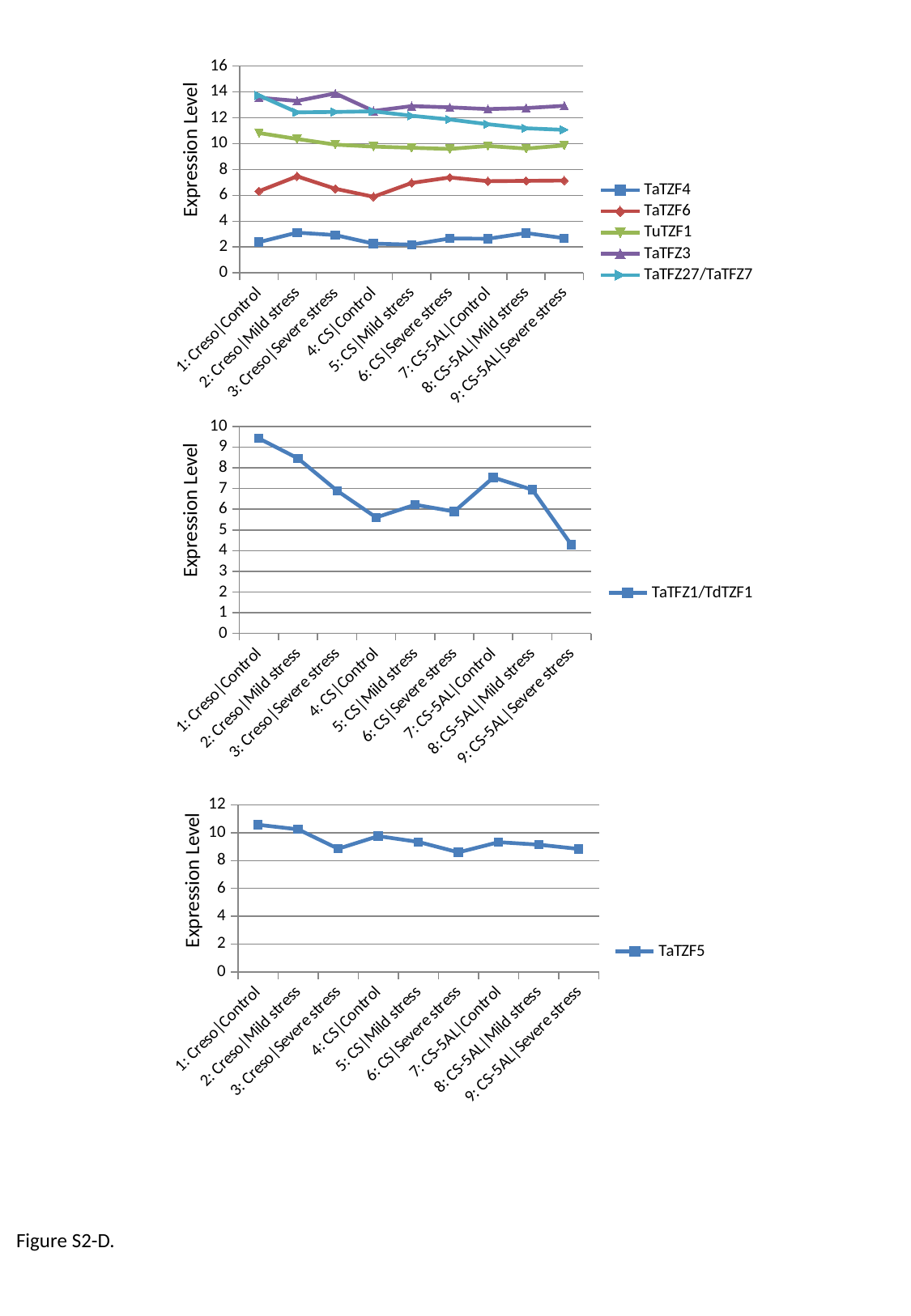

### Chart
| Category | TaTZF4 | TaTZF6 | TuTZF1 | TaTFZ3 | TaTFZ27/TaTFZ7 |
|---|---|---|---|---|---|
| 1: Creso|Control | 2.381182080924 | 6.325836392587965 | 10.82221347051067 | 13.55116629975402 | 13.72828610829433 |
| 2: Creso|Mild stress | 3.1214929179759 | 7.474146566256167 | 10.3615652390837 | 13.30298883908704 | 12.42208223484337 |
| 3: Creso|Severe stress | 2.929172831766133 | 6.510949290733342 | 9.922703161011965 | 13.88316652062502 | 12.45183937107802 |
| 4: CS|Control | 2.265950622507471 | 5.891942272056634 | 9.771604904689866 | 12.52837251313433 | 12.50024998841334 |
| 5: CS|Mild stress | 2.190869918861 | 6.96036430298877 | 9.68065923828427 | 12.90245732166636 | 12.15572568389133 |
| 6: CS|Severe stress | 2.667869864501035 | 7.385998761368967 | 9.59150963722799 | 12.80471100032 | 11.86546966884704 |
| 7: CS-5AL|Control | 2.643839158019867 | 7.09600555880913 | 9.81815497867067 | 12.67435072790033 | 11.49959438112202 |
| 8: CS-5AL|Mild stress | 3.08272469574234 | 7.123842898014638 | 9.619998104973867 | 12.74893910982767 | 11.18556173688034 |
| 9: CS-5AL|Severe stress | 2.686397085720905 | 7.148751923725766 | 9.859332023021855 | 12.9255950759697 | 11.06344065188203 |Expression Level
### Chart
| Category | TaTFZ1/TdTZF1 |
|---|---|
| 1: Creso|Control | 9.421313677420825 |
| 2: Creso|Mild stress | 8.45653273778777 |
| 3: Creso|Severe stress | 6.897395532304234 |
| 4: CS|Control | 5.604972975846477 |
| 5: CS|Mild stress | 6.218986950693087 |
| 6: CS|Severe stress | 5.8904462140891 |
| 7: CS-5AL|Control | 7.533174691171968 |
| 8: CS-5AL|Mild stress | 6.946696405949412 |
| 9: CS-5AL|Severe stress | 4.282615350204067 |Expression Level
### Chart
| Category | TaTZF5 |
|---|---|
| 1: Creso|Control | 10.57691583975868 |
| 2: Creso|Mild stress | 10.24734393846181 |
| 3: Creso|Severe stress | 8.849188612049353 |
| 4: CS|Control | 9.760457126320903 |
| 5: CS|Mild stress | 9.341032456321772 |
| 6: CS|Severe stress | 8.593773255998032 |
| 7: CS-5AL|Control | 9.324869097082567 |
| 8: CS-5AL|Mild stress | 9.144383676876725 |
| 9: CS-5AL|Severe stress | 8.8366404662993 |Expression Level
Figure S2-D.

## Slide 6
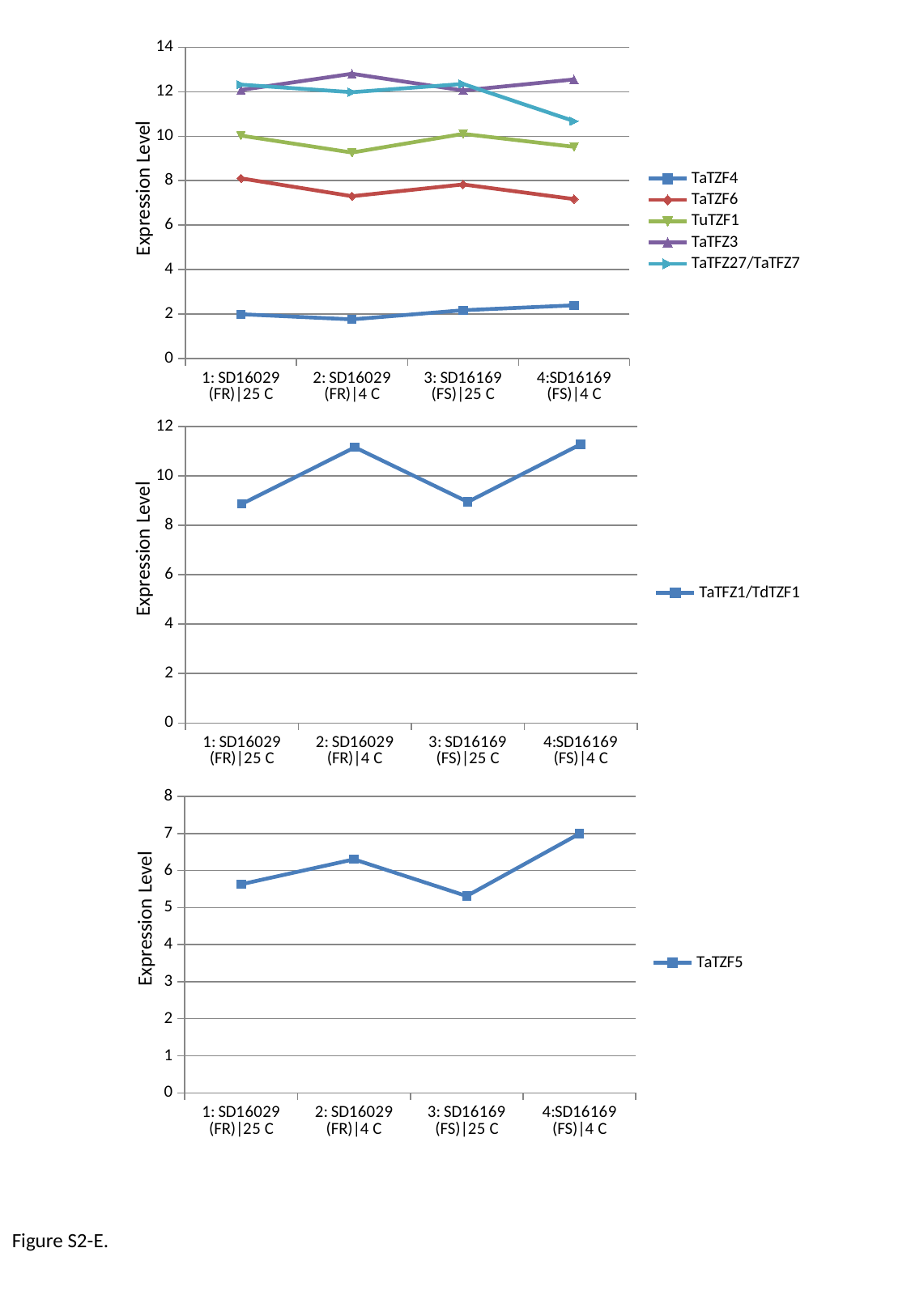

### Chart
| Category | TaTZF4 | TaTZF6 | TuTZF1 | TaTFZ3 | TaTFZ27/TaTFZ7 |
|---|---|---|---|---|---|
| 1: SD16029 (FR)|25 C | 1.990152036374303 | 8.107386391715501 | 10.02938499636406 | 12.08150224770651 | 12.32053250742952 |
| 2: SD16029 (FR)|4 C | 1.7578196632216 | 7.3020544254407 | 9.265075710730587 | 12.81266282883902 | 11.981309352827 |
| 3: SD16169 (FS)|25 C | 2.169639238508301 | 7.831222494846768 | 10.10744992729565 | 12.05909452736252 | 12.3533172746765 |
| 4:SD16169 (FS)|4 C | 2.39048742594235 | 7.1718579625245 | 9.524018715802848 | 12.55633884566201 | 10.6796557295695 |Expression Level
### Chart
| Category | TaTFZ1/TdTZF1 |
|---|---|
| 1: SD16029 (FR)|25 C | 8.86588055344238 |
| 2: SD16029 (FR)|4 C | 11.15654949767602 |
| 3: SD16169 (FS)|25 C | 8.947337774012132 |
| 4:SD16169 (FS)|4 C | 11.2805079900775 |Expression Level
### Chart
| Category | TaTZF5 |
|---|---|
| 1: SD16029 (FR)|25 C | 5.62836971063685 |
| 2: SD16029 (FR)|4 C | 6.301614650367437 |
| 3: SD16169 (FS)|25 C | 5.31020158451555 |
| 4:SD16169 (FS)|4 C | 6.992024222408196 |Expression Level
Figure S2-E.
