# Supplementary figures and images for: Conservation of AtTZF1, AtTZF2, and AtTZF3 homolog gene regulation by salt stress in evolutionarily distant plant species
Source: Front Plant Sci. 2015 Jun 16;6:394. doi: 10.3389/fpls.2015.00394 (PMC4468379; doi:10.3389/fpls.2015.00394)

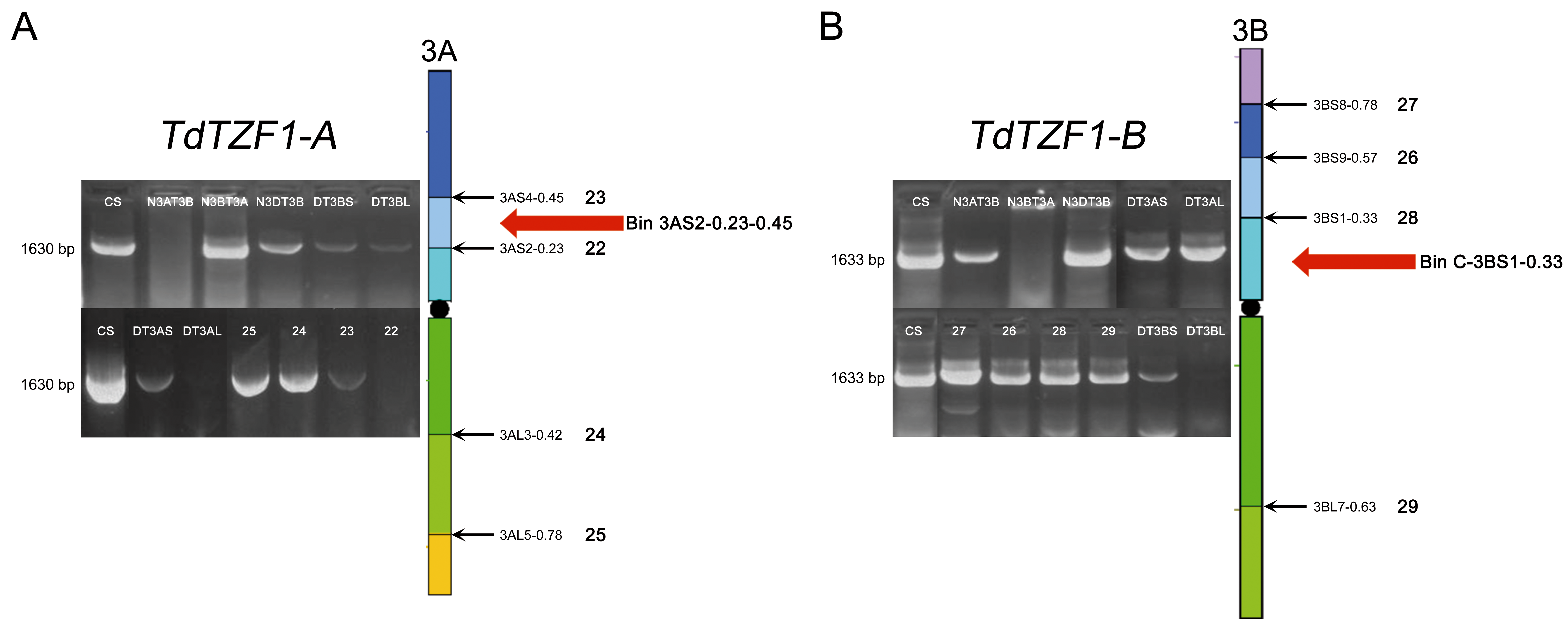

Supplement: Supplementary file 1 [file Supplementary_Materials.ZIP › correct files/132224_Morelli_Supplementary_Figure_S1.TIF]

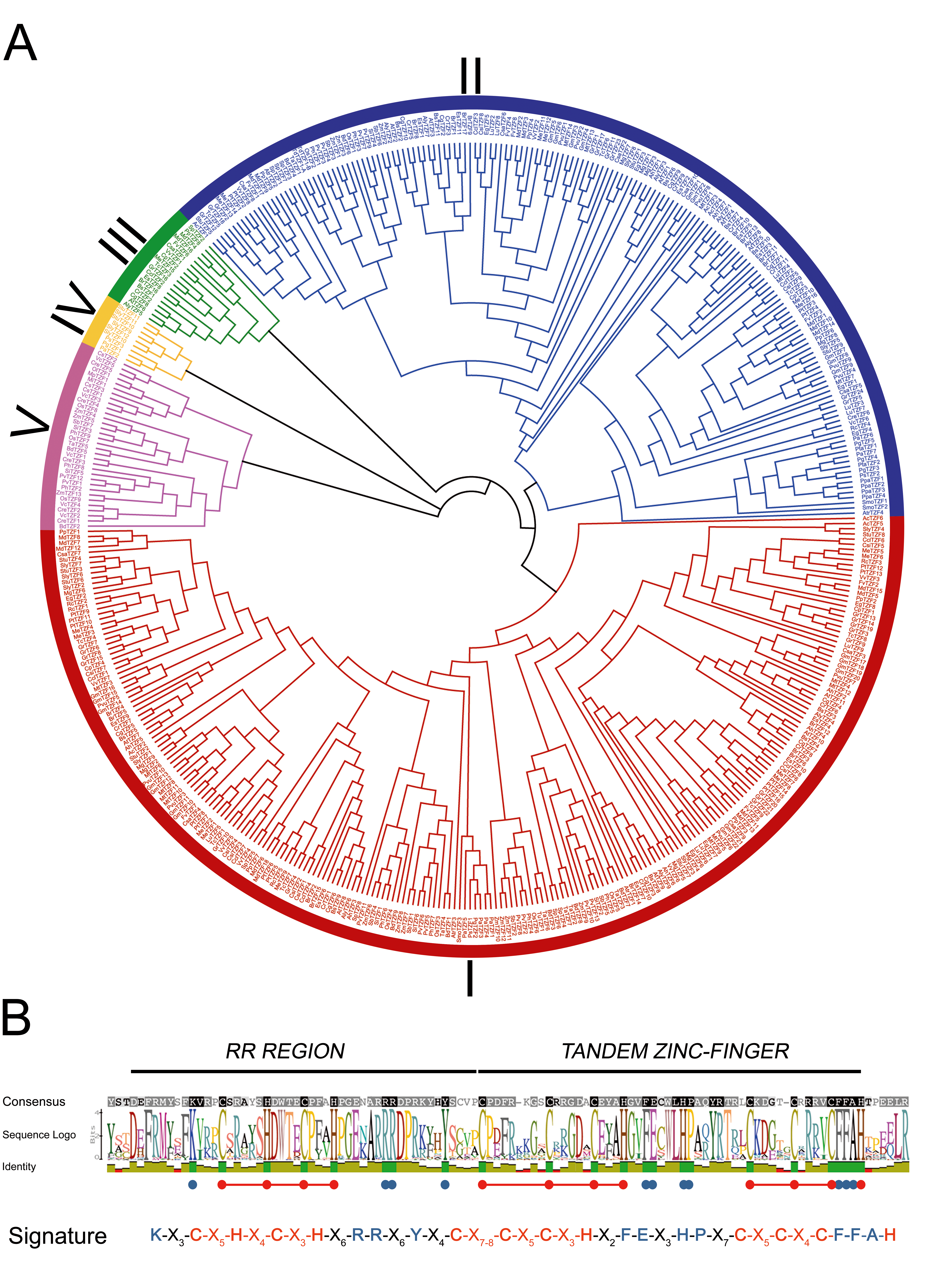

Supplement: Supplementary file 1 [file Supplementary_Materials.ZIP › correct files/132224_Morelli_Supplementary_Figure_S3.TIF]

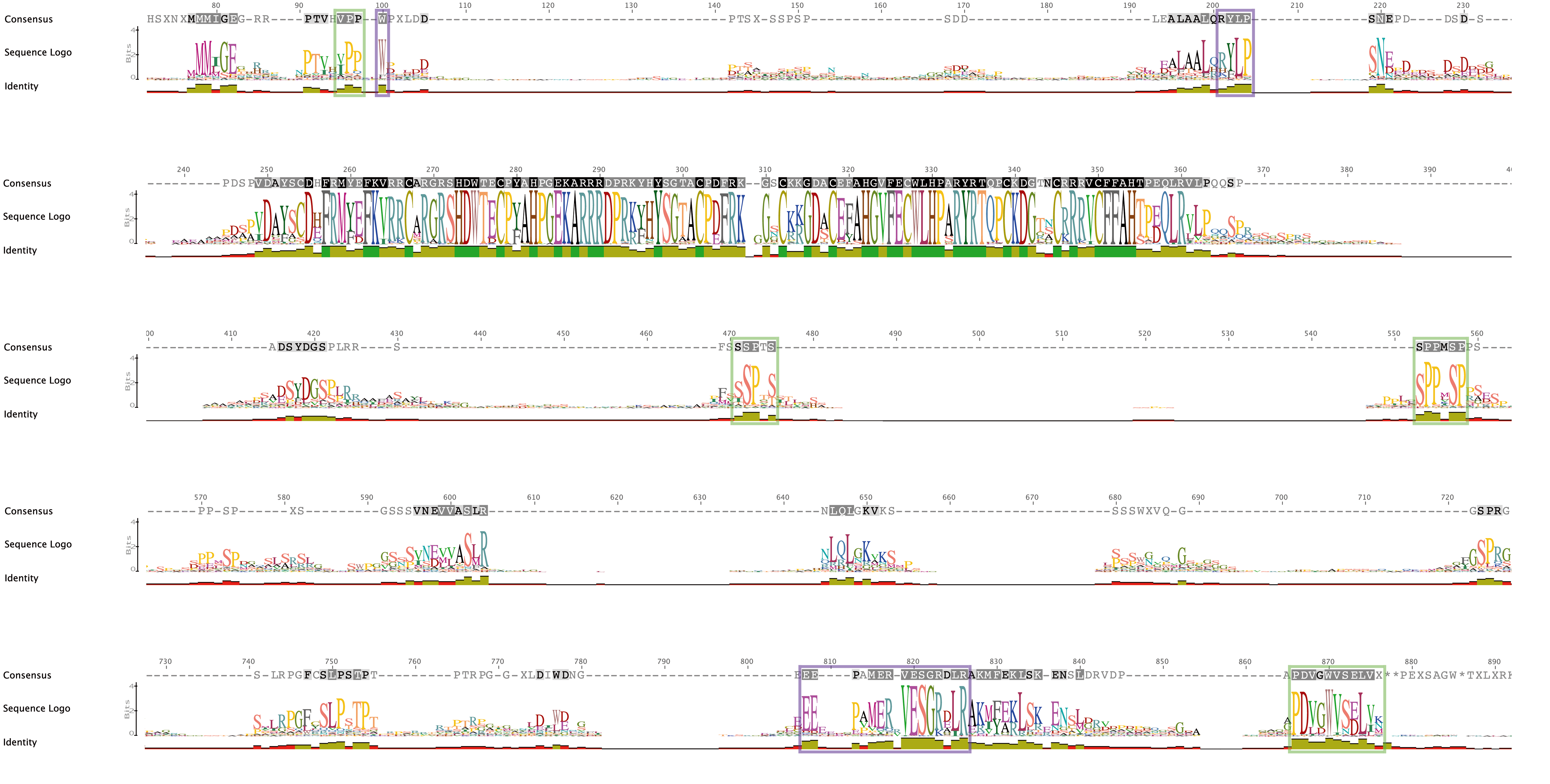

Supplement: Supplementary file 1 [file Supplementary_Materials.ZIP › correct files/132224_Morelli_Supplementary_Figure_S4.TIF]
